# Engaging and supporting the public on the topic of grief and bereavement: an evaluation of Good Grief Festival

**DOI:** 10.1177/26323524231189523

**Published:** 2023-07-30

**Authors:** Lucy E. Selman, Nicholas Turner, Lesel Dawson, Charlotte Chamberlain, Aisling Mustan, Alison Rivett, Fiona Fox

**Affiliations:** Palliative and End of Life Care Research Group, Population Health Sciences, Bristol Medical School, University of Bristol, 39 Canynge Hall, Bristol BS8 2PS, UK; Bristol Population Health Science Institute and Bristol Medical School, University of Bristol, Bristol, UK; Department of English, School of Humanities, Faculty of Arts, University of Bristol, Bristol, UK; Palliative and End of Life Care Research Group, Population Health Sciences, Bristol Medical School, University of Bristol, Bristol, UK; Wild Thing Productions Ltd, Bristol, UK; Public Engagement, University of Bristol, Bristol, UK; Population Health Sciences, Bristol Medical School, University of Bristol, Bristol, UK

**Keywords:** bereavement, death literacy, evaluation, grief literacy, public engagement

## Abstract

**Background:**

Good Grief Festival was originally planned as a face-to-face festival about grief and bereavement. Due to COVID-19, it was held online over 3 days in October 2020.

**Objective:**

To evaluate the festival’s reach and impact.

**Design:**

Pre/post evaluation

**Methods:**

Pre-festival online surveys assessed reasons for attending and attitudes to bereavement across four items (fear of saying the wrong thing, avoiding talking to someone bereaved, knowing what to do if someone bereaved was struggling, knowing how to help). Post-festival online surveys evaluated audience experiences and the four attitude items. Free-text responses, analysed using thematic analysis, generated suggestions for improvement and general comments.

**Results:**

Between 5003 and 6438 people attended, with most attending two to five events. Pre-festival survey participants (*n* = 3785) were mostly women (91%) and White (91%). About 9% were from Black or minoritised ethnic communities. About 14% were age ⩾65 years, 16% age ⩽34 years. Around 75% were members of the public, teachers, students or ‘other’; 25% academics, clinicians or bereavement counsellors. A third had been bereaved in the last year; 6% had never been bereaved. People attended to learn about grief/bereavement (77%), be inspired (52%) and feel part of a community (49%). Post-festival participants (*n* = 685) reported feeling part of a community (68%), learning about grief/bereavement (68%) and being inspired (66%). 89% rated the festival as excellent/very good and 75% agreed that they felt more confident talking about grief after attending. Higher ratings and confidence were associated with attending more events. Post-festival attitudes were improved across all four items (*p* < 0.001). Attendees appreciated the festival, particularly valuing the online format, opportunities for connection during lockdown and the diversity and quality of speakers. Suggestions included improving registration, more interactive events and less content.

**Conclusion:**

Good Grief Festival successfully reached a large public audience, with benefit in engagement, confidence and community-building. Evaluation was critical in shaping future events. Findings suggest festivals of this nature can play a central role in increasing death- and grief-literacy within a public health approach.

## Plain language summary

What was the impact of Good Grief Festival?

**Why was this study done?** We designed Good Grief Festival to improve knowledge about bereavement and support for bereaved people. Due to the pandemic, the festival was held online over 3 days in October 2020. In this study, we assessed the festival’s impact on the people who attended.

**What did the researchers do?** Before the event, we sent an online survey to everyone who registered. It asked their attitudes to bereavement, such as whether they were ‘scared of saying the wrong thing’ to bereaved people or knew how to help/support them. After the festival, we sent everyone who attended a survey containing the same pre-event questions, alongside extra questions about their experience of the festival and suggestions for improvement.

**What did the researchers find?** Between 5005 and 6438 people attended the festival. A total of 3785 people completed the pre-festival survey. They wanted to learn about grief/bereavement (77%), be inspired (52%) and feel part of a community (49%). The post-festival survey was completed by 685 people who said they learnt about grief/bereavement (68%), were inspired (66%) and felt part of a community (68%). Most people (89%) rated the festival as excellent/very good and said that through attending the festival they felt more confident in talking to a bereaved person and knowing how to support them. People who attended more events were more confident. Suggestions to improve the festival included improving registration, more interactive and arts-based events and offering a smaller programme overall. Most people who completed the survey were women (91%) and White (91%).

**What do the findings mean?** Overall, Good Grief Festival successfully reached a large public audience. It helped to improve people’s confidence when supporting bereaved people and made them feel part of a community. The survey feedback helped us improve and shape future events.

## Introduction

A key aspect of a public health approach to palliative and end-of-life care is supporting communities to have conversations and learn more about death, dying, grief and bereavement. Such initiatives often aim to improve death and grief literacy, while strengthening existing community-based resources and providing individuals with the benefit of social support and connection.^1,2^ We designed and delivered ‘Good Grief Festival’ in line with this approach. The festival aimed to support discussion and learning around grief and bereavement while also widening access, both to multi-disciplinary research and scholarship and to formal and informal bereavement support. In this sense, the aims of the festival were centred on both public engagement and reducing inequities in access to information and support. Here, we report an evaluation of the festival’s reach and impact to inform future public engagement and public health initiatives in palliative and end-of-life care and bereavement.

### Public engagement

‘Public engagement’ refers to the myriad of ways in which the activity and benefits of higher education and research can be shared with the public. It is increasingly recognised as an essential aspect of academic research and is by definition a two-way process, involving interaction and listening, with the goal of generating mutual benefit.^3^ The growth of public engagement is in part due to a recognition that historically ‘ivory tower’ academic research deprived much of the public of the insight and knowledge, and therefore the power and societal benefit, which can come from research evidence and scholarship. In particular, disadvantaged or structurally vulnerable populations are less likely to participate in, access or engage with academic research,^4^ with some arguing that racism is evident across the UK health research landscape.^5^

### Health inequities in bereavement and palliative and end-of-life care

There are well-evidenced inequities in access to health and social care and other formal support services, including discrimination and a lack of sensitivity to the requirements of diverse minoritised and under-served population groups. This includes palliative and end-of-life care, and bereavement and mental health support. Bereavement sector policies^6–10^ mandate equity and fair access, yet there is evidence that certain population groups are less likely to proactively seek out and access professional care and support – even when needed and wanted – and are more likely to feel uncomfortable asking for help.^11,12^ The lesbian, gay, bisexual, transgender, queer or questioning and more (LGBTQ+) communities experience barriers to accessing bereavement support as well as additional stressors, for example, discrimination, homophobia, disenfranchisement, historical illegality and higher rates of social isolation.^13^ Barriers to access faced by minoritised ethnic communities include: experiences of institutional racism (including in healthcare); a lack of awareness of bereavement support (often due to poor information provision by professionals^14^ and a lack of outreach by services); culturally or individually inappropriate support, and, for some minoritised communities, additional stigma regarding mental health.^15^ Specific groups of bereaved people may be disadvantaged and disenfranchised in multiple ways, due to varied dimensions of their structural vulnerability, with gender, class and age acting as additional, intersecting axes of inequity.^16^ In addition, bereavement itself can constitute a form of social inequity, exposing grieving individuals to policy, processes, systems and networks that function in disenfranchising ways,^16^ for example, by promoting ‘productivity’ and ‘stoicism’.^17^

### Public health approaches to bereavement

Public health approaches to bereavement recommend a tiered approach based on level of need.^18,19^ Tier 1 includes universal access to information on grief and available support, recognising that many bereaved people cope without formal intervention, drawing on their existing social networks. Tier 2 includes individual and group-based support for those with moderate needs (estimated at 30%), who have been shown to benefit from increased social support and opportunities for reflection, emotional expression and restorative activities. Tier 3, that is, specialist mental health and psychological support is effective for those with high-level needs (estimated at 10%) and at risk of prolonged grief disorder and should be targeted at those who meet these criteria.^18,20,21^ Information and community sources of support, therefore, play an essential role for everyone who experiences a bereavement, with approximately 60% of bereaved people relying solely on this level of support.^18,19^

However, as well as experiencing barriers to accessing Tier 2 and 3 formal support, bereaved people can struggle to get the information and support they need at a Tier 1 level. For example, in a study of people bereaved during the first 9 months of the COVID-19 pandemic in the United Kingdom, just 34% of participants had been given any information about bereavement support after the death.^14^ While support from family and friends was used by 89% of bereaved participants and perceived as helpful, 39% reported difficulties getting support from friends and family, with a fifth feeling uncomfortable asking for help.^22^ Barriers such as a lack of understanding and compassion among family and friends, and difficulties expressing feelings and needs after bereavement, also existed pre-pandemic^23–26^ and continue to be reported: a 2022 representative UK survey found 43% of adults worried about saying the wrong thing to someone bereaved, 32% did not know how to start a conversation after a bereavement, and 12% had gone out of their way to avoid someone who is grieving because they do not know what to say to them.^27^ Among bereaved people, 60% said their community had not helped them deal with their grief and 30% reported people not referencing their bereavement.^27^ Similarly, in a survey by Sue Ryder, 79% of bereaved people reported that informal support did not sufficiently meet their needs.^28^ More needs to be done to increase understanding, normalise conversations about death, dying and bereavement, and break down barriers to accessing both formal and informal support – as recently recognised by the UK Commission on Bereavement.^29^

### Good Grief Festival

Festivals are an increasingly popular way to engage the public in science and research,^30^ offering an opportunity to engage directly with researchers, scholars and other ‘experts’ via an event that provides a brief and concentrated focus on a specific topic. Good Grief Festival was developed to serve a dual aim of engaging people in research and scholarship related to grief, bereavement and end-of-life care across diverse disciplines, and to widen access to support and information regarding bereavement, giving people more confidence in talking about grief. We aimed to evaluate the reach and impact of the festival to inform future public engagement initiatives on this topic.

## Methods

### Study design

Pre-/post open online surveys were disseminated to everyone who registered to attend the first Good Grief Festival, which was held from 30 October to 1 November 2020. No incentives were given for survey completion. The evaluation framework was informed by a recent systematic review of the evaluation of public health festivals.^31^ Reporting follows the Checklist for Reporting Results of Internet E-Surveys.^32^

#### Design of the festival

A city-wide face-to-face festival about grief and bereavement was planned to take place in Bristol, UK, in May 2020. Due to the pandemic, the event was postponed to October 2020 and transformed into an online event. Our aims were to provide ways for people to talk, think, share and learn about experiences of grief, and to widen access to research. We did this through co-designing the festival with a large group of collaborators from cultural institutions, community organisations and charities, and members of the public. The co-design process involved discussions with diverse groups about the content, advertising and delivery of the festival and regular meetings to shape the festival, plan and discuss progress (three meetings with project partners and collaborators, three meetings with a local bereavement network, two meetings with clinicians, counsellors and academics, two meetings with diverse groups of adults with experiences of care and bereavement and one meeting with a group of young people between the ages of 11 and 18). Partnerships with organisations including Bristol Black Carers (https://www.bristolblackcarers.org.uk/) and the BAMEStream Alliance – an alliance of therapists, policy specialists, organisations, activists and academics across the United Kingdom, which was established during the COVID-19 pandemic to ‘bring the mental health needs of the Black, Asian and Minority Ethnic community into the mainstream’ – aimed to ensure the festival speakers and facilitators were diverse and the programme was inclusive.

Over the 3 days of the online festival (30 October to 1 November 2020), a programme of panel discussions, interviews, creative events, webinars and workshops, covered a wide range of topics related to grief. The festival was structured to include several components: panel discussions, talks and interviews with high-profile contributors; Grief School educational sessions with people with lived experience and academics; Grief Chat sessions between two people with similar experiences of loss; participatory sessions such as yoga and deaths cafes; and webinars and workshops on topics such as creating meaningful funerals and memorials and supporting someone who is bereaved. Some of the panel discussions and interviews were live while others were prerecorded and live streamed during the festival. All the Grief School and Grief Chat sessions were available to view on demand throughout the festival. The full programme is available as Supplementary file (S1).

The festival was free to attend and open to all. In the 2 weeks after the festival, all content was captioned and uploaded to the Grief Channel, which served as a repository for Good Grief events. The Grief Channel cost £20 a year and provided on-demand access to the festival events. The festival was funded through a grant from the Wellcome Trust.

### Data collection

#### Surveys

A voluntary pre-event online survey was developed to assess the festival’s audience characteristics and reasons for attending and tested by team members (see Supplementary file S2). The survey opened 2 months before the festival (when pre-registration for festival events opened). Based on previous polling by Sue Ryder,^11^ participants were asked to rate their level of agreement with statements about their attitudes towards interactions with those who are bereaved. Participants could respond: strongly agree, tend to agree, tend to disagree, strongly disagree or don’t know.

A voluntary post-event survey (see Supplementary file S3) assessed experiences of the festival and was open for 3 weeks following the festival. Participants were asked to rate their experience of attending the festival on a 5-point Likert-type scale (0 = poor to 5 = excellent), and to indicate their level of agreement with the statement ‘through attending the festival I feel more confident talking about grief’. Participants were also asked the same attitudinal questions as in the pre-event survey. The post-festival questionnaire also included two free-text items: ‘Please tell us your suggestions for how the festival could have been improved’ and ‘Is there anything else you would like to say about the festival?’

The surveys were created in SurveyMonkey and disseminated with a request for feedback via emails to attendees, the festival’s social media, a blog on the festival website and during the festival in the online chat. Festival attendees were requested to complete the survey only once, regardless of how many events they attended, and the survey was set to allow one response per participant using the same device. There was no financial remuneration for participating.

### Analysis

Quantitative analysis was conducted by a statistician (NT) and qualitative analysis by a qualitative health services researcher (FF). Neither data analyst was part of the team which designed and delivered the festival.

#### Quantitative analysis

Participant characteristics were summarised using descriptive statistics, categorical data were presented as frequencies and percentages and continuous data as mean and standard deviation, or median and interquartile range (dependent on the nature of the distribution of the variable).

For the pre-festival survey, reasons for attendance were analysed: the number (and proportion) of participants who selected each option are reported. Each of these options was treated as a binary outcome identifying if that was or was not a reason for attendance, and entered in (separate) logistic regression models investigating the association between demographic characteristics (age category, gender, ethnicity, UK residency and what audience group they most identify as), and experience of bereavement, with reason for attendance. Participant rating of level of agreement with attitudes towards interacting with those who are bereaved was used as the outcome in an ordinal regression model to investigate the association between participant characteristics and level of agreement with the statements. Those who responded ‘don’t know’ were not included in these analyses.

Post-festival questionnaire data regarding participants attendance and experiences of the festival is presented descriptively both as ‘raw’ unweighted data and weighted, to make the post-festival sample data more representative of the pre-festival population. Inverse probability weighting via logistic regression was used to generate the weights. Rating of festival experience was used as a continuous outcome variable in linear regression to investigate the association between rating of experiences and participant characteristics and festival attendance data. The variable ‘confidence talking about grief’ was used as the outcome in ordinal regression and associations with participant characteristics and festival attendance explored.

Data from the pre- and post-festival surveys regarding attitudes about interacting with those who are bereaved were combined and ordinal regression used to investigate if level of agreement with the statements differed dependent on whether participants were responding pre- or post-festival. Data were also compared with that from the 2019 Sue Ryder national survey.^11^

#### Qualitative analysis

Free-text survey data were analysed using inductive thematic analysis^33^ in NVivo v12 (FF). Preliminary themes were discussed with LES, however due to LES’s involvement in delivering the festival, FF managed the data analysis process and coded the responses alone.

## Results

### Reach – attendance, media and social media

Over the three days on the festival, the festival was attended by 5003 individuals (unique viewers) who accessed the livestreamed festival on Vimeo; in addition, Room 2 webinars had a total of 1435 participants. It is unknown how many webinar participants also watched the livestream and how many people attended more than one webinar; therefore, the total number of people who attended across the 3 days is between 5003 and 6438. This is a conservative estimate that does not include participants who watched with family or friends. From November 2020 until 22 March 2021, an additional 1800 individuals accessed recordings of the festival’s events on Vimeo via the Grief Channel (a second festival was held from 23 to 24 March 2021, not reported here).

A media report by the University of Bristol found a media reach of 1.22 billion via 72 articles syndicated around the world, reaching Shanghai and New Zealand. The festival built up a social media following of 14,000 across Twitter, Facebook and Instagram between September and November 2020.

### Participant characteristics

The pre-festival survey was completed by 3785 people and the post-festival survey by 685 people. Completeness rates were 100% and 96% for the pre- and post-festival surveys, respectively. Average time to complete was 1:59 and 3:57 min, respectively. Most participants in both the pre- and post-festival samples were female (91% and 90%, respectively; see Table 1). Most were White, defined themselves as members of the public, and were residing in the United Kingdom at the time of the questionnaires. The most common age categories in both samples were 45–54 and 55–64. The post-festival sample was a slightly older cohort than the pre-festival sample. A total 94% of participants had experienced a bereavement, with 33% participants experiencing a bereavement within the past year. A total of 341 participants responded to the free-text item requesting suggestions for how to improve the festival, and 402 participants to the item ‘is there anything else you would like to say?’

**Table 1.** Demographic characteristics of pre- and post-festival samples.

Characteristic	Pre-festival sample (*N* = 3785)	Post-festival sample (*N* = 685)
	*N* (%)	*N* (%)
Age group (years)		
Under 18	3 (<1)	1 (<1)
18–24	83 (2)	9 (1)
25–34	499 (1)	58 (8)
35–44	727 (1)	122 (18)
45–54	1016 (26)	167 (24)
55–64	988 (26)	197 (29)
65–74	463 (12)	121 (18)
⩾75	76 (2)	12 (2)
Gender		
Female	3480 (91)	615 (90)
Male	332 (9)	64 (9)
Prefer to think of yourself in another way	30 (1)	6 (1)
Ethnicity		
White	3498 (91)	626 (92)
Mixed/Multiple ethnic groups	119 (3)	19 (3)
Asian/Asian British	107 (3)	21 (3)
Black/African/Caribbean/Black British	39 (1	5 (1)
Other ethnic group	66 (2)	13 (2)
Currently reside in the United Kingdom (Yes)	2838 (74)	612 (89)
Description of self		
Member of the public	1700 (44)	265 (39)
Bereavement counsellor	462 (12)	96 (14)
Academic interested in grief	159 (4)	31 (5)
Clinician	330 (9)	38 (6)
Teacher	108 (3)	19 (3)
Student	196 (5)	25 (4)
Other	888 (23)	213 (31)
Has participant experienced the death of a relative, partner or close friend (otherwise known as bereavement)?		
Yes, within the last year	1290 (33)	–
Yes, within the last 5 years	1347 (35)	–
Yes, more than 5 years ago	1002 (26)	–
No	214 (6)	–

### Reasons for attending

Reasons for attending the festival are presented in Table 2; the most frequent reasons were to learn about grief and bereavement (77%), to be inspired (52%), and to feel part of a like-minded community (49%).

**Table 2.** Reason for attending festival (pre-festival sample, *N* = 3785).

Characteristic	*N* (%)
Reason for attending festival (participants able to tick multiple options)	
Be inspired	2012 (52)
Feel part of a like-minded community	1901 (49)
Share or express experiences	1072 (28)
Learn about grief and bereavement	2950 (77)
Find out about local bereavement support	541 (14)
Other	539 (14)
Number of reasons for attending the festival	2 [1, 3]^a^

a Median [interquartile range (IQR)].

The results of the logistic regression models exploring attending for specific reasons based on demographic characteristics are presented in Supplementary Table S1 and described below.

#### Be inspired

There was strong evidence that those in the 35–44 age category had greater odds of attending the festival to be inspired, compared with the grand mean of the age categories [odds ratio, OR 1.56 (1.10, 2.20)]. There was also strong evidence of a difference in odds in attending the festival to be inspired dependent on how participants self-described: those identifying as bereavement counsellors, academics interested in grief, clinicians, students or ‘other’ had greater odds of attending to be inspired compared with members of the public. There was evidence that those who had experienced bereavement more than 5 years ago or within the last 5 years (but not within the last year) had greater odds of attending the festival to be inspired, while those who had experienced bereavement within the last year had reduced odds of attending to be inspired, compared with the grand mean of the categories. There was no evidence of a difference in odds based on gender, ethnicity or residence in the United Kingdom.

#### Feel part of a like-minded community

There was evidence that whether participants attended the festival to feel part of a like-minded community differed by audience group. Clinicians were less likely to attend to feel part of a like-minded community, compared with members of the public [OR 0.71 (0.55, 0.90)]. There was also evidence that those who had experienced a bereavement were more likely to have attended the festival to feel part of a like-minded community than those who had not experienced a bereavement. There was no evidence of association with any other participant characteristic.

#### Share or express experiences

There was a strong ‘dose–response’ relationship whereby the more recently someone was bereaved, the greater the odds of attending the festival to share or express experiences. There was strong evidence that those identifying themselves as bereavement counsellors, academics interested in grief, clinicians or students had lower odds of attending for the purpose of sharing or expressing experiences compared with members of the public.

#### Learn about grief and bereavement

Those who had experienced a bereavement were less likely to attend the festival to learn about grief and bereavement than those who had not. Those identifying students were more likely to attend to learn compared with members of the public [OR 2.43 (1.50, 3.96)]. Older participants were less likely than younger participants to attend to learn about grief and bereavement.

#### Find out about local bereavement support

Those currently residing in the United Kingdom had greater odds of attending to find out about local bereavement support compared with those not residing in the United Kingdom [OR 1.34 (1.07, 1.68)]. There was evidence of a ‘dose–response’ relationship whereby the more recently a participant had experienced a bereavement, the greater the odds of them attending to find out about local bereavement support.

### Post-festival evaluation

Most participants attended two to five festival events (42%), or several days (27%). A total 68% reported feeling part of a like-minded community, 68% learnt about grief and bereavement and 66% reported they were inspired (weighted data; see Table 3).

**Table 3.** Post-festival evaluation (*N* = 685).

Characteristic	Un-weighted	Weighted
	*N* (%)	(%)
Number of events attended		
I did not attend in the end	30 (4%)	5%
1 or 2	162 (24%)	24%
2–5	291 (42%)	42%
A whole day	18 (3%)	2%
Several days	188 (27%)	27%
At the event(s) participant . . . (able to tick multiple options)		
Was inspired	466 (67%)	66%
Felt part of a like-minded community	468 (68%)	68%
Shared or expressed experiences	107 (16%)	16%
Learnt about grief and bereavement	464 (67%)	68%
Found out about local bereavement support	58 (8%)	9%
Other	113 (16%)	17%
Rating of overall experience (0 = poor to 5 = excellent)	5 [4, 5]	5 [4, 5]
Through attending the festival, participant feels more confident talking about grief		
Strongly agree	256 (38%)	37%
Tend to agree	250 (37%)	37%
Neither agree nor disagree	67 (10%)	11%
Tend to disagree	7 (1%)	1%
Strongly disagree	3 (<1%)	<1%
Don’t know	4 (1%)	1%
NA – already felt as confident as I could do	82 (12%)	12%

NA, not applicable.

The overall rating of festival experience was median 5 (excellent; interquartile range 4, 5), and this was reflected in the free-text data. The overwhelming majority of comments responding to the item ‘is there anything else you would like to say?’ were positive, with many respondents saying how grateful they were for the festival. The word ‘thanks’ was used by 30% of survey respondents. The festival was described as ‘great’ or ‘good’ by 27% and as ‘excellent’, ‘amazing’ or ‘wonderful’ by 21%. Participants particularly valued the quality of the speakers, the balance of informative and therapeutic sessions, and the opportunity to connect with others:

It was beautifully organised, an incredible mixture of therapeutic and informative sessions. All speakers were both incredibly knowledgeable about their topic, but also personally present and appropriately vulnerable and authentic in connecting with their own loss stories. The various means available for attendees to make their voices heard were helpful and hit just the right note to begin to feel like a community, to help us all feel a bit more ‘normal’. ID 12184614067

A surprisingly positive experience, exceptionally well organised, talented appropriate speakers and this made a positive difference to my present grieving situation but perhaps also future situations. ID 12128828171

I really think everything was superb – communications, organization, the quality of the speakers, moderators and the technology. I would attend again for sure. ID 12135079645

From the lens of having organized large events, all I can offer is kudos. I was wowed at how the festival transcended so many barriers not only in content but in creating safe, communal spaces within a cyber platform. Also was amazed that your team was able to not let the festival get out of hand, i.e., become a platform for societal grievances. And, appreciated with wisdom behind the timing (e.g., in sync with All Saints Day, etc.) of the festival. ID 12135092639

Most thought that the online format was successful, with several participants remarking that they would have not been able to attend had it been a live event (either due to distance from Bristol or the emotional nature of the topic):

It was a very good festival and I learnt a lot about my own grief. In one way it was good to have the event online as this made it easier for me to attend. I am not sure I would have the courage to attend in person. ID 12133844520

Keep it online. I would not have travelled to Bristol for this I would have been too emotionally challenged to travel home afterwards so being available in the safety of home was excellent. ID 12129055796

While it was acknowledged that in-person events allow greater interaction, many felt that this would be too confronting, given the topics being covered.

### Factors associated with rating of experience

Results of linear regression investigating factors associated with rating of festival experience are presented in Supplementary Table S2. The only variable for which there was evidence of an association with rating of experience was number of festival sessions. A greater number of festival events attended was associated with a higher rating of experience.

### Confidence

A total 74% reportedly agreed that the festival had helped them feel more confident talking about grief. There was evidence that a greater number of sessions attended was associated with increased odds of agreeing more strongly with the statement ‘through attending the festival I feel more confident talking about grief’. Those who identified as a bereavement counsellor, clinician or other were more likely to agree more strongly with this statement compared with members of the public. Full results of ordinal regression investigating factors associated with level of agreement with the statement that participants felt more confident having attended the festival are presented in Supplementary Table S3.

### Attitudes towards someone recently bereaved

Levels of agreement with the four attitude statements are presented in Supplementary Table S4 for the nationally representative data from the Sue Ryder survey, the pre-festival sample and the post-festival sample. There was strong evidence (*p* < 0.001) that there was a difference in the level of agreement between participants from the Good Grief Festival and the (population weighted) Sue Ryder survey sample for each statement. Participants from the Good Grief Festival were less likely to agree that they would be scared of saying the wrong thing to someone recently bereaved and that they would avoid taking to someone recently bereaved about their bereavement, and were more likely to agree that they would know what kind of help or support to offer if someone who was recently bereaved told them they were struggling. In the pre-festival sample (*n* = 3785), men were significantly less likely than women to say they would know what kind of help to support or offer someone who was bereaved (*p* = 0.003; see Table S6). There was a linear relationship between some attitudes and age, with older people less likely to be scared of ‘saying the wrong thing’ (*p* < 0.001) and less likely to avoid talking to someone bereaved because they would not know how to help (*p* < 0.001; see Supplementary Table S5).

Pre- and post-festival attitudes to the bereaved are compared in Table 4. There was evidence that those in the post-festival sample were less likely to be in a higher category of agreement with the statements ‘I would be scared of ‘saying the wrong thing’ to someone who was recently bereaved’ and ‘I would avoid talking to someone who was recently bereaved about their bereavement because I wouldn’t know how to help’ than those in the pre-festival sample. Compared with those in the pre-festival sample, those in the post-festival sample where more likely to be in a higher category of agreement with the statements ‘I would know what to do if someone who was recently bereaved told them they were having trouble’ and ‘I would know what kind of help or support to offer someone who was bereaved’.

**Table 4.** Comparison of pre- and post-festival attitudes with the bereaved, adjusted for age, gender, ethnicity, residence, description of self.

	pOR (95% CI)	*p*-value
Participant would be scared of ‘saying the wrong thing’ to someone who was recently bereaved (*N* = 4372)		
Pre-festival sample	Ref	
Post-festival sample	0.49 (0.42, 0.57)	< 0.001
Participant would avoid talking to someone who was recently bereaved about their bereavement because they would not know how to help (*N* = 4374)		
Pre-festival sample	Ref	
Post-festival sample	0.63 (0.53, 0.75)	< 0.001
Participant would know what to do if someone who was recently bereaved told them they were having trouble (*N* = 4245))		
Pre-festival sample	Ref	
Post-festival sample	1.88 (1.60, 2.23)	< 0.001
Participant would know what kind of help or support to offer someone who was bereaved (*N* = 4225)		
Pre-festival sample	Ref	
Post-festival sample	1.98 (1.68, 2.35)	< 0.001

CI, confidence interval; OR, odds ratio.

## Suggestions for improvement

Suggestions for improving aspects of the festival were extracted from responses to the free-text item ‘is there anything else you would like to say about the festival?’. These related to: advertising, meeting needs of both professionals and the public, the registration process, the online format, the content (volume, duration, speakers/facilitators, topics for future events), diversity, support, interactivity and creative and well-being events. Findings are summarised in Table 5, along with an indication of whether and how these suggestions helped shape subsequent Good Grief events held in 2021/2022.

**Table 5.** Suggestions for improvement.

Theme	Participants’ views and suggestions, with example quotes	Incorporation into future Good Grief events
Advertising	• Wider publicity, especially internationally. • Colour scheme difficult for some, for example, ‘I found the use of stark black and white graphics very difficult to look at, especially during talks. I have some sensory issues and would have found it an easier experience with softer graphics’ (ID 12192074513) – however, benefits were also reported, for example, regarding the gender neutral design.	Increased investment in social media advertising. Simplification of the audience view by using Zoom rather than a branded green screen background.
Registration	• A total 22% found the registration process hard to navigate, time-consuming or confusing. • Greater clarity in the programme regarding the content of and the target audience for each event; in particular, participants wished to know in advance whether workshops were interactive. • Some found the volume of email reminders too high.	Simplification of registration process and addition of FAQs page. Improved labelling and description of events to improve clarity.
Format	• Suggestion that for future events a ‘hybrid model’ would be best. This would allow the option to attend in person, whilst also making the festival accessible to a wider international audience. • A small number of people cited uncertainty over how to access the live content, or technical difficulties. • People would have liked immediate access to sessions when they ended, or the ability to download them soon after the live streaming. • A small number felt that the £20 fee to access all festival content for a year was prohibitive.	Good Grief Festival now has a large international audience. To continue to support everyone without excluding people who do not live in a specific area, the festival is continuing with an online model. In May 2022, the first in-person festival was held in Weston-super-Mare, in collaboration with a network of community organisations (see: https://cultureweston.org.uk/journals/good-grief-weston-the-films/). The Good Grief archive is being moved to the YouTube channel (@griefchannel), launched September 2022, providing free on-demand access.
Content – volume, duration	• About 6% of survey responses indicated that there was too much content over the weekend. ‘maybe just suggest people sign up to a few as a taster if they are not doing on demand as I wanted to join too many and then couldn’t keep up with it all as hadn’t fully considered the emotional impact. There was so much to process’ (ID 12128405691) • Suggestions of a mix of sessions of different events and running shorter festivals/events spread over a longer period. ‘Life is pretty hectic and I did want to listen to/take part in so much more, but just couldn’t fit half as much into the week with work and a toddler around. Maybe it could be spread over a longer period of time?’ (ID 12169853852) • Recognition that it can be challenging to meet the needs of both the public and professionals. ‘I think the professionals can take the onslaught of information and talk about death (and how people died), but for those who are grieving it can be really overwhelming so maybe some safeguards put in place for the lot who are actually going through the process. I did love the festival though because it was the first time in 13 years that I felt I finally found a place to explore what I’d been through and meet with others who’ve had a similar experience.’ (ID 12172505543)	Good Grief Festival moved to a ‘mini-festival’ format from January 2022: fewer but more regular events over a day rather than many events over a weekend. The Good Grief archive is being moved to the YouTube channel (@griefchannel), launched September 2022, with specific themed playlists enabling on-demand open access.
Content – speakers/facilitators	• Some deemed the quality of individual speakers and session Chairs as ‘variable’. • Some disliked speaker ‘self-promotion,’ such as selling their books.	Ongoing curation of speakers and facilitators.
Content – topics for future events	Abortion grief Ambiguous loss / other kinds of loss Children’s grief – age specific Dementia experience Funeral industry Grief as a cancer patient Losing a parent No touch death Non-finite loss Pre-death grief Sibling bereavement • To augment the events, downloadable resources could be provided, such as resources for bereavement counsellors or key quotes from the talks	Many of these topics were incorporated into subsequent festivals and mini-festivals in 2021 and 2022. The new Good Grief Hub website (www.goodgriefhub.org, launched spring 2023) incorporates additional resources and sign-posting, with the YouTube channel (@griefchannel) providing curated playlists of specific topics and themes.
Diversity & inclusion	• There was an appetite for sessions covering more diverse faith perspectives, cultural issues and class, as well as provision for the hearing impaired, or those with sensory impairments. ‘The festival was excellent and the only suggestion I have is that maybe there could have been more focus on working class people’s experiences of grief. Many speakers and panellists spoke from a middle class position and perhaps experienced grief differently to those of us who don’t have the same resources (and therefore headspace because we are struggling to afford to live). People speaking about grieving against a backdrop of other struggles would have felt more relatable to me’ (ID 12133850003)	All Good Grief events are captioned before being made available on-demand. The festival continues to emphasise diversity in the topics and speakers included.
Support	• Some highlighted the need for support during or after the festival, suggesting that this could be provided via: • Self-care reminders; • Allocated time for reflection processing between sessions; • More links to grief support services and counselling. ‘I would suggest that you have people on standby to help, a listening ear, in chat or online.’ (ID 12143603599)	Good Grief Festival has a well-developed Resources webpage and festival staff direct people to resources and give self-care reminders during all sessions. The new Good Grief Hub (www.goodgriefhub.org) increases sign-posting to services and support and includes links to access online and text support.
Interactivity	• Greater interaction in small groups and workshops, with opportunities to share personal experiences and stories. • Some struggled to work out how to post on the chat board and a few found it distracting. • Extend the live chat after events, enable connection between audience members. • Make it clear which events are pre-recorded and which are not, which events include interactivity and which do not. ‘I could see that some people were making connections on the live chats. Could there be a facility for them to connect at the end of the event if they wanted to as you would do in non- digital settings? It felt like an abrupt ending when the sessions finished as people were still having grief conversations.’ (ID 12130063022)	Improved labelling and description of events to improve clarity. Exploring options to connect the audience via a Forum.
Creative and well-being events	• Many participants wanted a greater degree of creative sessions that were either performative or interactive to allow attendees time and space to process difficult emotions that may have been raised during other festival sessions. • In particular, participants wanted more provision of yoga, music, film or other performing arts to balance out the sessions based on talking and discussion.	Inclusion of well-being events such as yoga and creative writing as well as regular sessions on creativity and nature and grief, and a short film series in May 2022.

## Discussion

This evaluation of the first Good Grief Festival held in October 2020 demonstrates that between 5003 and 6438 people engaged with the event over the 3 days, with pre/post data providing preliminary evidence that it helped raise confidence and improve attitudes to bereavement. Overall, we saw an increase in confidence in about three-quarters of participants and significant improvement in attitudes post-festival (with the proviso that post-festival data were not from the same sample), despite better attitudes to bereavement among festival attendees compared with a national sample. The festival successfully engaged the general public: just 25% (pre- and post-festival samples) of attendees were bereavement counsellors, academics or clinicians, with the remainder members of the public, teachers, students or ‘other’.

The festival evaluation surveys were completed by a much larger proportion of women (91% pre-festival sample; 90% post-festival) than men. The gender bias in responses is likely to reflect a lack of engagement with survey completion as well as lower attendance levels of men at the festival. A similar gender bias in other public health initiatives on this topic and in bereavement research has been previously reported.^14,34,35^ A scoping review of gender disparities in end-of-life care found that women were overall more able to express needs and ask for help, while men tended to try to be stoic and independent rather than voice symptoms or feelings.^36^ Similarly, different coping styles have been reported in bereavement: using the Dual Process conceptual model, women appear to be more loss-oriented following bereavement, feeling and expressing their distress at their loss; men more restoration-oriented, actively engaging with the problems and practical issues associated with loss.^37,38^ In qualitative focus groups with male participants (to be published separately), we explored views and acceptability of the festival in more detail.

Non-White participants made up 9% of the pre-festival sample and 8% post-festival. This is similar to other public engagement events on this topic,^34^ but not nationally representative (82% of people in England and Wales and 96% in Scotland are White). By building on the evaluation presented here and continuing to focus on equity and representativeness we were able to increase this to 10% non-White participants at the second festival. The festival continues to focus on diversity in speakers, facilitators and content in an effort to ensure inclusivity and widen access, and in 2022 launched a YouTube Channel^39^ to provide free access to festival content.

Good Grief Festival is one of a small number of UK festivals specifically focused on death, bereavement or grief, and is perhaps unique in its prioritisation of research and scholarship. Published post-event reports or evaluations of such festivals are rare, although there are some good examples.^40^ This general absence is likely due to a lack of resources for collection and analysis of feedback, or a lack of awareness that others might find such reports useful. A number of festivals^41,42^ operate on a highly participative basis – with the festival organisers arranging a small number of headline activities and undertaking promotion, while most events are conceptualised and delivered by individuals and organisations on their own initiative – which might further complicate the process of evaluation. Festival organisers might also prioritise more qualitative or creative representations of a festival, for example, in the form of a short film, over a formal evaluation.

Evaluations and reports in the public sphere indicate that other similar festivals or engagement activities attract in-person audiences of several hundred to several thousand, depending on their exact nature and duration.^34,35,40,43,44^ Attendance at Good Grief 2020 was higher than most at over 5000, although the figures are not necessarily comparable as Good Grief was entirely online and took place during the COVID-19 ‘lockdown’ period when many people were keen to attend virtual events. Our high engagement figures reflect a strong appetite for online events, which were largely acceptable and preferred by many participants, particularly given the emotive subject matter. Reported advantages to the festival being online included widening access, being able to engage in private from home, and being able to dip in and out of sessions, providing individual control. However, these data are from participants who attended an online festival and completed an online evaluation; online events will not be acceptable, accessible and preferred by everyone, so offering diversity in types of public engagement events on this topic is important.

While the aims, foci and methodologies of the few publicly available evaluations vary, they find similar outcomes for attendees. Participants are generally positive about their involvement; they appreciate the chance to talk openly about grief and bereavement; think it is important to have opportunities for conversations around death, dying and loss; and often find it helpful to share their own experiences, wishes and fears or to be assisted with end-of-life planning.^34,35,40^

The study has several implications: our findings can help shape future engagement initiatives internationally by demonstrating novel methods of engaging people on the topic of grief and bereavement and suggesting diverse topics for inclusion (see Table 5). Our methods for evaluating the festival can also inform future evaluation of public engagement festivals^31^ and death/grief literacy^45^ initiatives. The evaluation data provided useful evidence to help improve subsequent festivals; at the second festival in March 2021, evaluation ratings improved further: 92% rated it as excellent or very good and 80% agreed or strongly agreed with the statement ‘Through attending the festival I feel more confident talking about grief’ (compared with 89% and 76%, respectively at the first festival), and the audience became more ethnically diverse, underlining the importance of quality evaluation.

Study strengths include a relatively large sample, collection of qualitative free-text data as well as quantitative data, and the integration of the Sue Ryder attitudinal items, which enabled comparison with population-level data. A limitation of the evaluation is response bias: we only received responses from a proportion of people who attended, and it is possible that people with more favourable experiences, or who were more critical, were more likely to respond. Similarly, the quantitative analysis was conducted on a complete case basis; missing data within survey responses has the potential to introduce bias into findings. All events and surveys were only provided in English, therefore limiting engagement and feedback to those able to understand and write in English. Before uploading recorded festival events to the Grief Channel, captioning was added to all videos to increase accessibility; however, this was not available during live events. A total 94% of festival attendees indicated that they had experienced a bereavement, 33% in the last year. We did not quantitatively assess the impact of the festival on their bereavement or coping with grief, although free-text data indicated benefit in this area.

Further research is needed to assess the contribution of grief festivals to Tier 1 and 2 bereavement support and the ripple effects of this on bereavement experiences and outcomes as well as social attitudes. Novel methodologies are needed to map, describe and evaluate societal and systems change when considering population/country-wide initiatives. There is currently limited bereavement research in low- and middle-income country settings. This work lends evidence and opportunity for a public health festival approach to grief to be applied and evaluated in other contexts, including where Tier 1 and 2 bereavement support may be less accessible, for example. Other areas for future research include more in-depth examination of reach, impact and participant experiences of grief festivals to help include and benefit as many different population groups as possible. To this end, we also conducted in-depth focus groups with specific attendee groups (findings to be reported separately). Related to this, further research is needed to explore and explain the gender bias we found in the Good Grief Festival audience, which has also been reported in other public health and community initiatives on similar topics.^46^ Finally, research is needed to understand the perspectives and experiences of people who could not or would not access a festival of this type and what kind of engagement and/or community-building activities are more appropriate and acceptable in this group.

In conclusion, the online Good Grief Festival successfully reached and engaged a large public audience, with participants reporting that they felt inspired, part of a like-minded community and learnt about grief and bereavement. Three-quarters of participants reported that the festival increased their confidence talking about grief, with attitudes to supporting bereaved people better in the post-festival sample than in the pre-festival sample. These findings suggest festivals of this nature can play a central role in increasing death- and grief-literacy within a public health approach.^47^ Given the urgent need to reclaim death, dying and grief as social concerns; normalise open discussion of these topics; and bolster networks of care;^48^ we hope the festival and the findings of this evaluation encourage other large-scale public-facing initiatives to reduce inequities in access to knowledge, information, and support. By continuing this work and joining with others, we hope to help create the social movement that is needed.

## Supplemental Material

sj-docx-1-pcr-10.1177_26323524231189523 – Supplemental material for Engaging and supporting the public on the topic of grief and bereavement: an evaluation of Good Grief Festival Click here for additional data file. Supplemental material, sj-docx-1-pcr-10.1177_26323524231189523 for Engaging and supporting the public on the topic of grief and bereavement: an evaluation of Good Grief Festival by Lucy E. Selman, Nicholas Turner, Lesel Dawson, Charlotte Chamberlain, Aisling Mustan, Alison Rivett and Fiona Fox in Palliative Care and Social Practice

sj-docx-2-pcr-10.1177_26323524231189523 – Supplemental material for Engaging and supporting the public on the topic of grief and bereavement: an evaluation of Good Grief Festival Click here for additional data file. Supplemental material, sj-docx-2-pcr-10.1177_26323524231189523 for Engaging and supporting the public on the topic of grief and bereavement: an evaluation of Good Grief Festival by Lucy E. Selman, Nicholas Turner, Lesel Dawson, Charlotte Chamberlain, Aisling Mustan, Alison Rivett and Fiona Fox in Palliative Care and Social Practice

sj-pdf-3-pcr-10.1177_26323524231189523 – Supplemental material for Engaging and supporting the public on the topic of grief and bereavement: an evaluation of Good Grief Festival Click here for additional data file. Supplemental material, sj-pdf-3-pcr-10.1177_26323524231189523 for Engaging and supporting the public on the topic of grief and bereavement: an evaluation of Good Grief Festival by Lucy E. Selman, Nicholas Turner, Lesel Dawson, Charlotte Chamberlain, Aisling Mustan, Alison Rivett and Fiona Fox in Palliative Care and Social Practice

sj-pdf-4-pcr-10.1177_26323524231189523 – Supplemental material for Engaging and supporting the public on the topic of grief and bereavement: an evaluation of Good Grief Festival Click here for additional data file. Supplemental material, sj-pdf-4-pcr-10.1177_26323524231189523 for Engaging and supporting the public on the topic of grief and bereavement: an evaluation of Good Grief Festival by Lucy E. Selman, Nicholas Turner, Lesel Dawson, Charlotte Chamberlain, Aisling Mustan, Alison Rivett and Fiona Fox in Palliative Care and Social Practice

sj-pdf-5-pcr-10.1177_26323524231189523 – Supplemental material for Engaging and supporting the public on the topic of grief and bereavement: an evaluation of Good Grief Festival Click here for additional data file. Supplemental material, sj-pdf-5-pcr-10.1177_26323524231189523 for Engaging and supporting the public on the topic of grief and bereavement: an evaluation of Good Grief Festival by Lucy E. Selman, Nicholas Turner, Lesel Dawson, Charlotte Chamberlain, Aisling Mustan, Alison Rivett and Fiona Fox in Palliative Care and Social Practice

## Declarations

**Ethics approval and consent to participate:** This research was conducted according to the World Medical Association Declaration of Helsinki. Ethical approval to conduct the evaluation was granted by the University of Bristol Faculty of Health Sciences Research Ethics Committee [Ref: 111190]. Written informed consent to participate was obtained from participants.

**Consent for publication:** Written informed consent for publication was provided by the participants.

**Availability of data and materials:** Requests should be directed at the corresponding author.
