# Supplementary material for: Engaging and supporting the public on the topic of grief and bereavement: an evaluation of Good Grief Festival
Source: Palliat Care Soc Pract. 2023 Jul 30;17:26323524231189523. doi: 10.1177/26323524231189523 (PMC10392217; doi:10.1177/26323524231189523)
Supplement: sj-docx-1-pcr-10.1177_26323524231189523 – Supplemental material for Engaging and supporting the public on the topic of grief and bereavement: an evaluation of Good Grief Festival [file sj-docx-1-pcr-10.1177_26323524231189523.docx]

## Supplementary Tables

## Supplementary Table S1: Results of logistic regressions investigating factors associated with reasons for attending the festival

|  | **Be inspired (N=3785)** | | **Feel part of a like-minded community (N=3785)** | | **Share or express experiences (N=3785)** | | |  |
| --- | --- | --- | --- | --- | --- | --- | --- | --- |
|  | OR (95% CI) | p-value | OR (95% CI) | p-value | | OR (95% CI) | p-value | |
| Gender  *Female*  *Male*  *Think of yourself in another way* | Ref  1.06 (0.84, 1.34)  1.21 (0.57, 2.60) | 0.786 | Ref  0.80 (0.63, 1.01)  1.41 (0.65, 3.05) | 0.109 | | Ref  0.73 (0.56, 0.96)  1.22 (0.54, 2.75) | 0.073 | |
| Ethnicity  *White*  *Mixed/Multiple ethnic groups*  *Asian/Asian British*  *Black/African/Caribbean/Black British*  *Other ethnic group* | Ref  0.91 (0.63, 1.34)  1.36 (0.91, 2.05)  0.72 (0.37, 1.39)  0.84 (0.51, 1.38) | 0.405 | Ref  0.77 (0.53, 1.13)  1.02 (0.69, 1.53)  0.71 (0.37, 1.35)  0.81 (0.49, 1.33) | 0.481 | | Ref  0.94 (0.61, 1.45)  1.70 (1.11, 2.59)  0.84 (0.40, 1.76)  1.45 (0.85, 2.47) | 0.086 | |
| Currently reside in the UK (Yes) | 0.88 (0.75, 1.02) | 0.087 | 0.90 (0.77, 1.04) | 0.147 | | 0.97 (0.82, 1.14) | 0.693 | |
| Age Group  *Under 18*  *18-24*  *25-34*  *35-44*  *45-54*  *55-64*  *65-74*  *≥75* | 0.34 (0.03, 3.91)  0.62 (0.38, 1.01)  1.03 (0.83, 1.30)  1.22 (1.00, 1.48)  Ref  1.00 (0.84, 1.20)  0.92 (0.73, 1.16)  0.56 (0.34, 0.92) | 0.014 | 0.65 (0.06, 7.33)  1.18 (0.73, 1.90)  1.27 (1.01, 1.59)  1.12 (0.96, 1.42)  Ref  0.98 (0.82, 1.17)  0.91 (0.73, 1.14)  0.69 (0.42, 1.12) | 0.095 | | 2.05 (0.18, 23.89)  0.55 (0.30, 1.02)  0.92 (0.71, 1.18)  0.89 (0.72, 1.12)  Ref  1.09 (0.90, 1.33)  1.04 (0.81, 1.34)  0.64 (0.35, 1.17) | 0.202 | |
| Description of self  *Member of the public*  *Bereavement counsellor*  *Academic interested in grief*  *Clinician*  *Teacher*  *Student*  *Other* | Ref  2.04 (1.63, 2.54)  1.45 (1.03, 2.04)  1.46 (1.14, 1.86)  0.87 (0.58, 1.30)  1.98 (1.44, 2.74)  1.92 (1.62, 2.29) | <0.001 | Ref  1.13 (0.91, 1.40)  0.72 (0.51, 1.01)  0.71 (0.55, 0.90)  0.75 (0.50, 1.11)  0.85 (0.62, 1.16)  1.15 (0.97, 1.37) | 0.002 | | Ref  0.64 (0.49, 0.82)  0.57 (0.37, 0.87)  0.69 (0.52, 0.92)  1.06 (0.70, 1.63)  0.56 (0.37, 0.84)  0.96 (0.80, 1.16) | <0.001 | |
| Has participant experienced the death of a relative, partner or close friend?  *No*  *Yes, more than 5 years ago*  *Yes, within the last 5 years*  *Yes, within the last year* | Ref  1.14 (0.84, 1.55)  1.20 (0.89, 1.62)  0.75 (0.55, 1.01) | <0.001 | Ref  2.42 (1.73, 3.39)  3.50 (2.51, 4.86)  2.75 (1.97, 3.83) | <0.001 | | Ref  2.40 (1.47, 3.91)  3.74 (2.32, 6.05)  4.19 (2.59, 6.78) | <0.001 | |

Supplementary Table S1 (continued)

|  | **Learn about grief and bereavement (N=3782)** | | **Find out about local bereavement support (N=3785)** | | **Other (N=3782)** | |
| --- | --- | --- | --- | --- | --- | --- |
|  | OR (95% CI) | p-value | OR (95% CI) | p-value | OR (95% CI) | p-value |
| Gender  *Female*  *Male*  *Think of yourself in another way* | Ref  1.23 (0.93, 1.64)  0.67 (0.30, 1.51) | 0.213 | Ref  0.65 (0.45, 0.95)  1.06 (0.36, 3.10) | 0.086 | Ref  0.90 (0.63, 1.28)  3.34 (1.45, 7.68) | 0.014 |
| Ethnicity  *White*  *Mixed/Multiple ethnic groups*  *Asian/Asian British*  *Black/African/Caribbean/Black British*  *Other ethnic group* | Ref  0.67 (0.44, 1.03)  1.10 (0.66, 1.84)  0.46 (0.23, 0.91)  1.20 (0.65, 2.25) | 0.064 | Ref  0.83 (0.47, 1.47)  0.87 (0.48, 1.59)  1.89 (0.88, 4.05)  1.33 (0.68, 2.58) | 0.401 | Ref  1.36 (0.81, 2.28)  0.33 (0.13, 0.83)  0.71 (0.25, 2.06)  1.87 (1.02, 3.43) | 0.020 |
| Currently reside in the UK (Yes) | 1.03 (0.87, 1.23) | 0.717 | 1.34 (1.07, 1.68) | 0.010 | 0.92 (0.74, 1.14) | 0.453 |
| Age group  *Under 18*  *18-24*  *25-34*  *35-44*  *45-54*  *55-64*  *65-74*  *≥75* | *(empty)*  0.74 (0.41, 1.34)  1.06 (0.81, 1.41)  1.20 (0.94, 1.54)  Ref  0.70 (0.57, 0.87)  0.69 (0.54, 0.90)  0.44, (0.27, 0.72) | <0.001 | 3.65 (0.31, 42.68)  1.15 (0.59, 2.26)  1.18 (0.86, 1.64)  1.14 (0.86, 1.53)  Ref  1.30 (1.00, 1.68)  1.24 (0.89, 1.72)  0.79 (0.35, 1.78) | 0.536 | *(empty)*  1.27 (0.64, 2.52)  0.93 (0.65, 1.32)  1.04 (0.77, 1.40)  Ref  1.05 (0.81, 1.36)  1.29 (0.94, 1.77)  1.43 (0.75, 2.72) | 0.590 |
| Description of self  *Member of the public*  *Bereavement counsellor*  *Academic interested in grief*  *Clinician*  *Teacher*  *Student*  *Other* | Ref  0.92 (0.72, 1.18)  1.04 (0.69, 1.55)  1.08 (0.81, 1.45)  0.90 (0.57, 1.43)  2.43 (1.50, 3.96)  1.16 (0.94, 1.41) | 0.013 | Ref  0.81 (0.58, 1.13)  0.86 (0.51, 1.47)  0.67 (0.45, 1.00)  1.11 (0.65, 1.91)  1.10 (0.70, 1.71)  1.14 (0.90, 1.44) | 0.182 | Ref  2.23 (1.64, 3.03)  1.62 (0.97, 2.69)  1.79 (1.24, 2.58)  0.65 (0.28, 1.50)  1.85 (1.16, 2.95)  3.27 (2.57, 4.17) | <0.001 |
| Has participant experienced the death of a relative, partner or close friend?  *No*  *Yes, more than 5 years ago*  *Yes, within the last 5 years*  *Yes, within the last year* | Ref  0.54 (0.36, 0.82)  0.61 (0.41, 0.91)  0.87 (0.58, 1.32) | <0.001 | Ref  1.57 (0.87, 2.81)  2.15 (1.22, 3.80)  2.91 (1.65, 5.13) | <0.001 | Ref  1.45 (0.91, 2.29)  1.06 (0.67, 1.68)  1.24 (0.78, 1.98) | 0.057 |

### Supplementary Table S2: Results of linear regression investigating factors associated with rating of festival experience

|  | **Rating of experience (N=653)** | | |
| --- | --- | --- | --- |
|  | Adj mean diff (95% CI) | p-value |  |
| Gender  *Female*  *Male*  *Think of yourself in another way* | Ref  -0.18 (-0.036, 0.004)  -0.37 (-1.05, 0.31) | 0.092 |  |
| Ethnicity  *White*  *Mixed/Multiple ethnic groups*  *Asian/Asian British*  *Black/African/Caribbean/Black British*  *Other ethnic group* | Ref  -0.16 (-0.48, 0.16)  0.10 (-0.22, 0.42)  0.16 (-0.51, 0.84)  -0.22 (-0.61, 0.17) | 0.580 |  |
| Currently reside in the UK (Yes) | -0.01 (-0.19, 0.17) | 0.939 |  |
| Age Group  *Under 18*  *18-24*  *25-34*  *35-44*  *45-54*  *55-64*  *65-74*  *≥75* | *empty*  0.002 (-0.49, 0.50)  -0.17 (-0.41, 0.07)  0.08 (-0.09, 0.24)  Ref  -0.07 (-0.22, 0.07)  -0.16 (-0.31, 0.002)  0.06 (-0.22, 0.34) | 0.138 |  |
| Description of self  *Member of the public*  *Bereavement counsellor*  *Academic interested in grief*  *Clinician*  *Teacher*  *Student*  *Other* | Ref  0.06 (-0.11, 0.22)  0.11 (-0.14, 0.36)  0.05 (-0.21, 0.31)  0.04 (-0.30, 0.37)  0.11 (-0.14, 0.35)  0.15 (0.01, 0.28) | 0.526 |  |
| Number of events attended  *I did not attend in the end*  *1 or 2*  *2-5*  *A whole day*  *Several days* | Ref  2.08 (0.90, 3.27)  2.14 (0.96, 3.31)  2.47 (1.27, 3.66)  2.42 (1.25, 3.60) | <0.001 |  |

### Supplementary Table S3: Results of ordinal regression investigating factors associated with level of agreement that participants feel more confident having attended the festival

|  | **More Confident (N=572)** | |
| --- | --- | --- |
|  | pOR (95% CI) | p-value |
| Gender  *Female*  *Male*  *Think of yourself in another way* | Ref  1.08 (0.62, 1.88)  0.51 (0.08 (3.08) | 0.731 |
| Ethnicity  *White*  *Mixed/Multiple ethnic groups*  *Asian/Asian British*  *Black/African/Caribbean/Black British*  *Other ethnic group* | Ref  0.57 (0.22, 1.52)  1.70 (0.65, 4.50)  1.08 (0.22, 5.42)  0.72 (0.19, 2.66) | 0.608 |
| Currently reside in the UK (Yes) | 1.35 (0.77, 2.35) | 0.297 |
| Age Group  *Under 18*  *18-24*  *25-34*  *35-44*  *45-54*  *55-64*  *65-74*  *≥75* | *empty*  1.18 (0.29, 4.82)  1.29 (0.68, 2.44)  1.13 (0.69, 1.86)  Ref  0.96 (0.61, 1.50)  0.73 (0.45, 1.21)  2.22 (0.58, 8.55) | 0.528 |
| Description of self  *Member of the public*  *Bereavement counsellor*  *Academic interested in grief*  *Clinician*  *Teacher*  *Student*  *Other* | Ref  2.35 (1.37, 4.02)  1.92 (0.86, 4.27)  2.25 (1.02, 4.94)  1.19 (0.47, 3.03)  1.86 (0.79, 4.39)  2.08 (1.40, 3.10) | 0.004 |
| Number of events attended  *I did not attend in the end*  *1 or 2*  *2-5*  *A whole day*  *Several days* | 0.55 (0.10, 2.99)  Ref  1.89 (1.24, 2.87)  2.39 (0.90, 6.38)  3.06 (1.93, 4.85) | <0.001 |

## Supplementary Table S4: Audience attitudes towards someone who was recently bereaved

| **Statement** | **Sue Ryder national data**  **(N=2189)**  **N (%)** | **Pre-festival (N=3785)**  **N (%)** | **Post-festival**  **(N=685)** | |
| --- | --- | --- | --- | --- |
|  |  |  | **Unweighted**  **N (%)** | **Weighted**  **N (%)** |
| Participant would be scared of ‘saying the wrong thing’ to someone who was recently bereaved  *Strongly agree*  *Tend to agree*  *Tend to disagree*  *Strongly disagree*  *Don’t know* | 258 (12%)  852 (39%)  616 (28%)  299 (14%)  130 (6%)* | 263 (7%)  1065 (28%)  1408 (37%)  1040 (27%)  69 (2%) | 12 (2%)  99 (15%)  280 (41%)  282 (41%)  9 (1%) | 2%  18%  41%  38%  1% |
| Participant would avoid talking to someone who was recently bereaved about their bereavement because they wouldn’t know how to help  *Strongly agree*  *Tend to agree*  *Tend to disagree*  *Strongly disagree*  *Don’t know* | 80 (4%)  478 (22%)  750 (34%)  657 (30%)  185 (8%) | 36 (1%)  288 (8%)  1238 (32%)  2218 (58%)  62 (2%) | 9 (1%)  17 (2%)  176 (26%)  472 (69%)  8 (1%) | 2%  3%  28%  66%  1% |
| Participant would know what to do if someone who was recently bereaved told them they were having trouble  *Strongly agree*  *Tend to agree*  *Tend to disagree*  *Strongly disagree*  *Don’t know* | 221 (10%)  894 (41%)  566 (26%)  157 (7%)  318 (15%) | 729 (19%)  1920 (50%)  708 (18%)  288 (8%)  196 (5%) | 217 (32%)  344 (50%)  63 (9%)  48 (7%)  10 (1%) | 29%  53%  10%  6%  1% |
| Participant would know what kind of help or support to offer someone who was bereaved  *Strongly agree*  *Tend to agree*  *Tend to disagree*  *Strongly disagree*  *Don’t know* | 184 (8%)  858 (39%)  619 (28%)  172 (8%)  323 (15%) | 731 (19%)  1904 (50%)  772 (20%)  228 (6%)  202 (5%) | 216 (32%)  348 (51%)  62 (9%)  39 (6%)  18 (3%) | 30%  52%  10%  5%  3% |

### * Sue Ryder survey also included option ‘Prefer not to say’. For each of the four items, 2% selected this option.

### Supplementary Table S5: Results of ordinal regression investigating factors associated with level of agreement with statements around attitudes to the bereaved (pre-festival sample, N=3785)

|  | **Scared of ‘saying the wrong thing’ (N=3712)** | | **Avoid talking to someone – wouldn’t know how to help (N=3713)** | | **Would know what to do if someone was having trouble (N=3585)** | | **Would know what kind of help or support to offer someone (N=3573)** | |
| --- | --- | --- | --- | --- | --- | --- | --- | --- |
|  | pOR (95% CI) | p-value | pOR (95% CI) | p-value | pOR (95% CI) | p-value | pOR (95% CI) | p-value |
| Gender  *Female*  *Male*  *Think of yourself in another way* | Ref  1.23 (0.99, 1.52)  1.12 (0.56, 2.26) | 0.167 | Ref  1.46 (1.16, 1.84)  0.94 (0.47, 2.01) | 0.006 | Ref  0.80 (0.64, 1.00)  1.13 (0.57, 2.27) | 0.143 | Ref  0.68 (0.54, 0.85)  1.12 (0.53, 2.35) | 0.003 |
| Ethnicity  *White*  *Mixed/Multiple ethnic groups*  *Asian/Asian British*  *Black/African/Caribbean/Black British*  *Other ethnic group* | Ref  1.00 (0.70, 1.43)  1.59 (1.09, 2.33)  1.55 (0.80, 3.00)  0.92 (0.58, 1.46) | 0.108 | Ref  1.25 (0.86, 1.82)  1.84 (1.25, 2.70)  1.86 (0.96, 3.59)  0.96 (0.57, 1.61) | 0.008 | Ref  0.94 (0.65, 1.35)  1.19 (0.81, 1.76)  0.88 (0.45, 1.69)  0.94 (0.57, 1.56) | 0.888 | Ref  0.97 (0.67, 1.40)  1.50 (1.02, 2.22)  0.84 (0.43, 1.62)  0.76 (0.45, 1.27) | 0.213 |
| Currently reside in the UK (Yes) | 1.12 (0.97, 1.28) | 0.111 | 1.12 (0.96, 1.30) | 0.163 | 0.94 (0.81, 1.08) | 0.368 | 0.92 (0.80, 1.07) | 0.292 |
| Age Group  *Under 18*  *18-24*  *25-34*  *35-44*  *45-54*  *55-64*  *65-74*  *≥75* | 1.44 (0.14, 14.86)  3.33 (2.12, 5.25)  1.95 (1.59, 2.40)  1.32 (1.10, 1.58)  Ref  0.77 (0.65, 0.91)  0.75 (0.61, 0.92)  0.75 (0.48, 1.16) | <0.001 | 3.41 (0.35, 33.52)  2.52 (1.59, 4.01)  1.46 (1.17, 1.82)  1.06 (0.87, 1.29)  Ref  0.86 (0.72, 1.04)  0.89 (0.70, 1.12)  0.87 (0.52, 1.46) | <0.001 | 1.63 (0.16, 16.86)  0.56 (0.35, 0.89)  0.81 (0.66, 1.00)  0.98 (0.81, 1.18)  Ref  0.87 (0.73, 1.04)  1.00 (0.80, 1.25)  0.72 (0.45, 1.14) | 0.099 | 0.70 (0.09, 5.38)  0.50 (0.31, 0.79)  0.68 (0.55, 0.85)  0.96 (0.79, 1.16)  Ref  0.87 (0.73, 1.04)  0.97 (0.78, 1.22)  0.73 (0.45, 1.18) | 0.004 |
| Description of self  *Member of the public*  *Bereavement counsellor*  *Academic interested in grief*  *Clinician*  *Teacher*  *Student*  *Other* | Ref  0.25 (0.20, 0.30)  0.66 (0.48, 0.91)  0.55 (0.44, 0.69)  1.28 (0.89, 1.84)  0.98 (0.73, 1.31)  0.44 (0.38, 0.52) | <0.001 | Ref  0.19 (0.15, 0.25)  0.49 (0.34, 0.69)  0.66 (0.52, 0.84)  1.17 (0.80, 1.71)  0.78 (0.57, 1.05)  0.43 (0.36, 0.52) | <0.001 | Ref  7.14 (5.69, 8.96)  1.59 (1.14, 2.22)  1.85 (1.45, 2.35)  1.67 (1.14, 2.45)  1.38 (1.02, 1.87)  1.98 (1.67, 2.34) | <0.001 | Ref  9.70 (7.71, 12.21)  1.73 (1.24, 2.41)  1.99 (1.56, 2.52)  1.42 (0.97, 2.08)  1.44 (1.06, 1.95)  2.47 (2.08, 2.93) | <0.001 |
| Has participant experienced the death of a relative, partner or close friend?  *No*  *Yes, more than 5 years ago*  *Yes, within the last 5 years*  *Yes, within the last year* | Ref  0.48 (0.36, 0.64)  0.42 (0.32, 0.56)  0.45 (0.34, 0.60) | <0.001 | Ref  0.59 (0.44, 0.81)  0.54 (0.40, 0.73)  0.62 (0.46, 0.84) | 0.001 | Ref  1.33 (0.99, 1.78)  1.63 (1.22, 2.17)  1.27 (0.95, 1.70) | 0.001 | Ref  1.70 (1.27, 2.28)  2.22 (1.67, 2.95)  1.68 (1.26, 2.23) | <0.001 |
