# Supplementary material for: Engaging and supporting the public on the topic of grief and bereavement: an evaluation of Good Grief Festival
Source: Palliat Care Soc Pract. 2023 Jul 30;17:26323524231189523. doi: 10.1177/26323524231189523 (PMC10392217; doi:10.1177/26323524231189523)

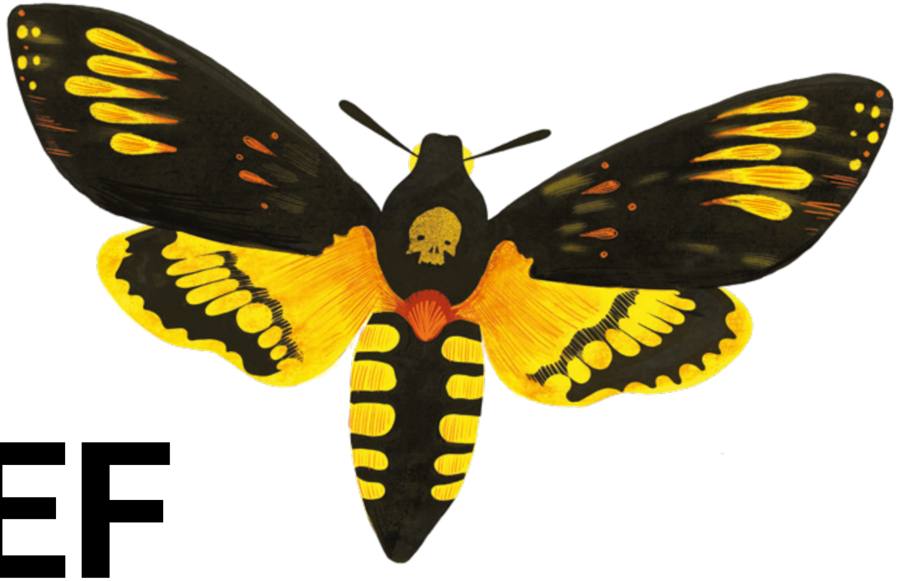

GOOD GRIEF

A FESTIVAL OF LOVE & LOSS

30 OCTOBER - 1 NOVEMBER 2020

BEST VIEWED ON DESKTOP OR PRINTED OUT

PROGRAMME

70+ EVENTS
100+ SPEAKERS
FREE EVENTS

WELCOME

Good Grief was originally planned as a week-long festival in Bristol to help people talk, think and learn about grief. But then…Covid-19 hit and it became clear that large scale live events won't be possible for some time.

Since then, we've been working hard to bring Good Grief Festival online as a global virtual event, taking place from October 30th to November 1st at a studio in the UK, reaching thousands of people all over the world.

We deeply believe that a festival exploring grief is now more important than ever before and we look forward to welcoming you virtually this October.

Many thanks,
The Good Grief Team

TICKETS

ON DEMAND ACCESS

£20

- Book festival events
- On demand access to recordings of all festival events for 3 years
- Subscription to The Grief Channel featuring 60+ hours of video content from the Good Grief Festival
- Watch live-streamed version of the festival
- Help to fund future Good Grief Events
- 20 CPD points from the Royal College of Physicians*

BUY NOW

ONE TIME ACCESS

FREE

- Book individual festival events
- One time access to live event

SIGN-UP

Terms + Conditions

- The £20 package entitles the user to view all recorded content from Good Grief Festival 2020 for 3 years. The content will be live on The Grief Channel two weeks after the festival. Login details will be sent when The Grief Channel is live.
- The user will also be able to view the festival as one live-streamed event. Login details will be sent out the week before the festival.
- The Federation of the Royal College of Physicians of the United Kingdom has approved Good Grief Festival for 20 category 1 (external) CPD credits, and archived access to this event for 20 external CPD points for 4 weeks after the event, application for approval beyond 4 weeks in progress.

wellcome

University of BRISTOL

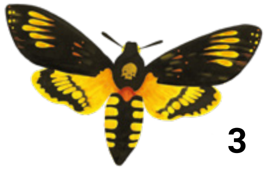

TALKS + INTERVIEWS

On the Main Stage, we have an incredible line-up of panel talks and interviews with speakers including Robert Webb, Cariad Lloyd, Valentine Warner, Julia Samuel, Professor Alice Roberts, Dr Rachel Clarke, Dr Kathryn Mannix and many, many more.
10:00 on Friday, October 30th to 19:00 on Sunday, November 1st.

GRIEF SCHOOL

Our Grief School will cover 14 different types of bereavement and loss including grief as a result of suicide, stillbirth, the death of a child, infertility, dementia, life-threatening illness diagnosis, pet loss, substance misuse, childhood bereavement and grieving during Covid-19.

Each Grief School event will repeat on a loop during the festival.

WORKSHOPS + WEBINARS

Our programme includes interactive workshops, and talks by academics across disciplines working on the topic of grief. Learn how to write a memoir with bestselling author Nikesh Shukla, design a meaningful memorial service with award-winning funeral celebrant Rosalie Kuyvenhoven or listen in to a lecture on Grief in Film with Dr Jimmy Hay.
09:30 on Friday, October 30th to 18:00 on Sunday, November 1st. The Studio.

GRIEF CHATS

Our Grief Chats are intimate conversations between two guests who have both experienced the same type of bereavement or loss. They are hosted by writer and broadcaster Mark Lemon, who’s behind the acclaimed podcast, Grief is my Superpower.

Each Grief Chat will repeat on a loop during the festival.

OUR SPEAKERS

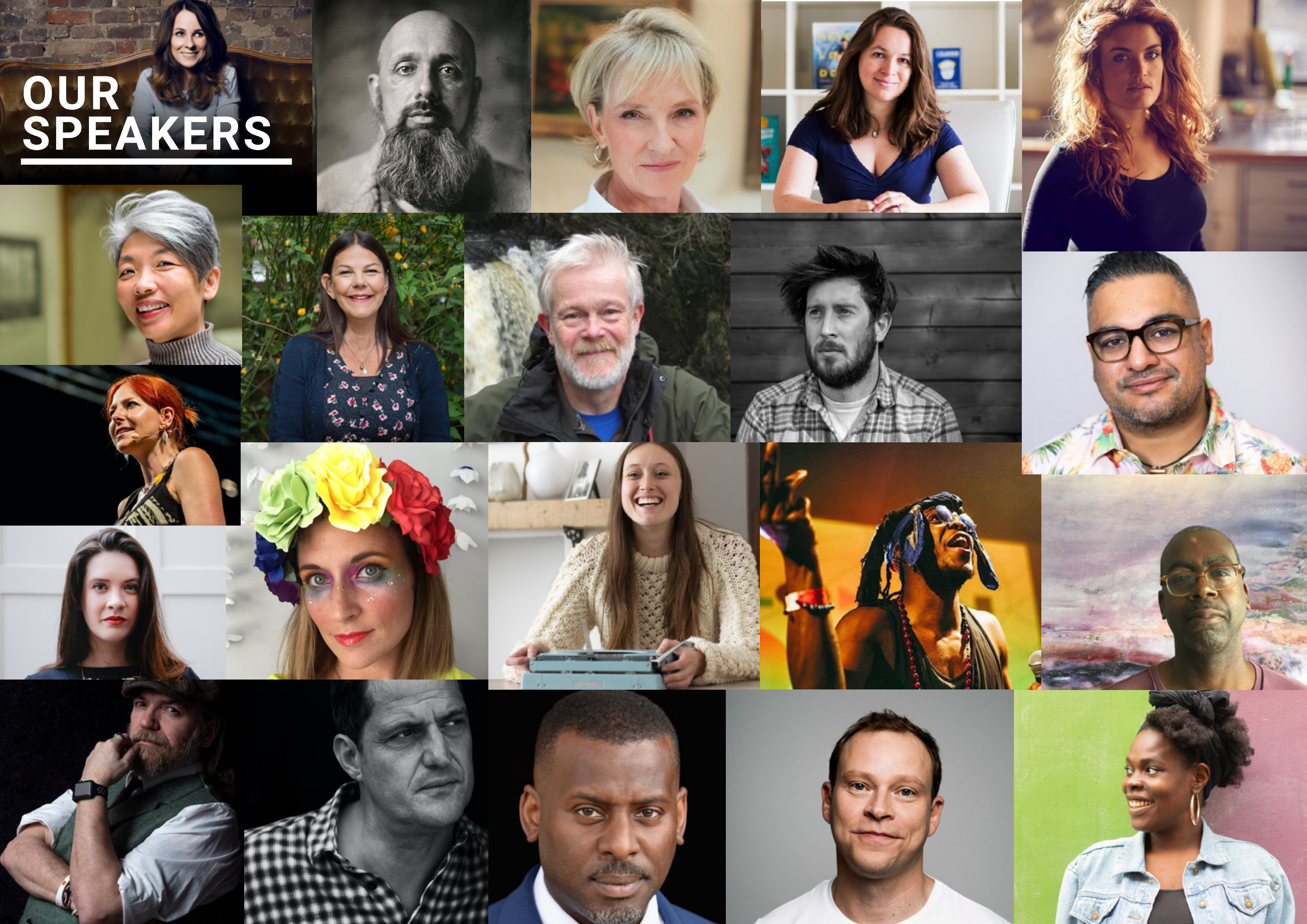

HIGHLIGHTS

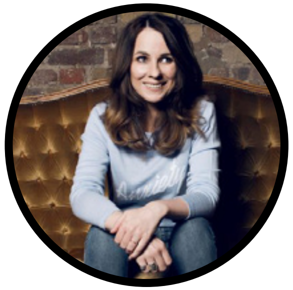

THE DEAD PARENT CLUB

Cariad Lloyd is the genius and voice behind Griefcast, the podcast in which funny people talk about death. In the Dead Parent Club, Cariad and guests will delve deep into what it means to lose a parent, how early bereavement is processed at different stages of life and how it's possible to find meaning that drives you forward.

SATURDAY, OCTOBER 31ST • 17:00-18:00

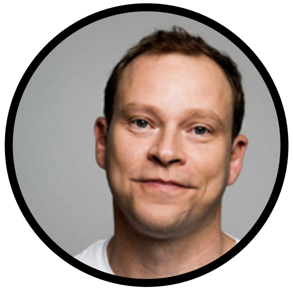

ROBERT WEBB

Robert Webb is an actor and comedian best known for That Mitchell and Webb Look and Peep Show. He's also a successful columnist and a bestselling author. Join us in conversation with Robert, who'll speak about his personal experience of losing a parent.

SATURDAY, OCTOBER 31ST • 18:00-19:00

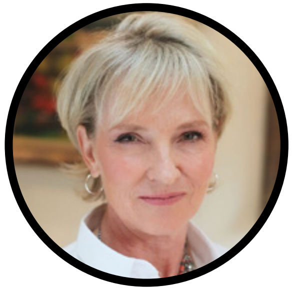

JULIA SAMUEL - COPING WITH GRIEF + CRISIS

In this free online session with the UK's leading grief expert, Julia Samuel MBE, you'll learn how to cope when your world falls apart. Julia will also discuss the transformative power of grief and loss and offer insight into how you can help those experiencing bereavement.

FRIDAY, OCTOBER 30TH • 17:00-18:00

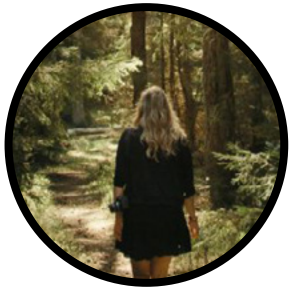

THE HEALING POWER OF NATURE IN GRIEF

Nature has an incredible ability to comfort and heal when we experience loss. It connects us with the cycles of the seasons and allows us to see the world, our lives, and ourselves in new ways. Join authors Long Litt Woon, Sarah Jane Douglas and Christopher Somerville as they share their experiences of turning to nature after bereavement.

SUNDAY, NOVEMBER 1ST • 15:30-16:30

GRIEF SCHOOL

Some people believe there are as many as 44 different types of grief. At our Grief School, we'll be delving into some of the ways we experience loss, so we can help people to better understand the nature of different types of bereavement and loss. Our Grief School will cover 14 different types of loss or bereavement including grief as a result of suicide, stillbirth, the death of a child, infertility, dementia, life-threatening illness diagnosis, pet loss, substance misuse, childhood bereavement and grieving during Covid-19.

FRIDAY, OCTOBER 30TH • FROM 09:00

GRIEF SCHOOL

HIGHLIGHTS

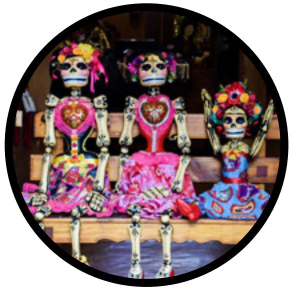

OVER MY DEAD BODY

Many of us have a deeply uncomfortable relationship with dead bodies. Join this enlightening talk with the BBC's Professor Alice Roberts, Dr John Troyer (Director of the Centre for Death and Society) and progressive funeral director, Louise Winter as they discuss the separation between the practical aspects of disposing of a dead body and the desire to grieve and memorialise a life.

SATURDAY, OCTOBER 31ST • 15:30-16:30

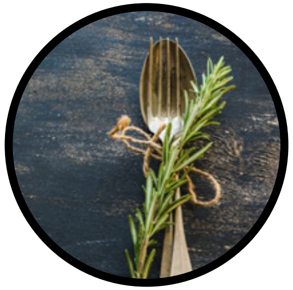

GRIEF AT THE KITCHEN TABLE

The kitchen table is often the epicentre of the grieving process. It's a backdrop for sadness, laughter and night-long conversations about loved ones who've died. It's where chairs remain empty at mealtimes – a poignant reminder of those who are no longer with us. In Grief at the Kitchen Table, writers Nikesh Shukla, Valentine Warner and Olivia Potts will consider how food can be a uniting factor in facing and moving through grief.

SATURDAY, OCTOBER 31ST • 14:30-15:30

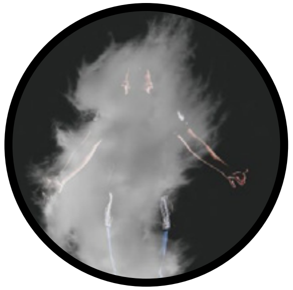

MAKING FRIENDS WITH YOUR AFTERLIFE

Societal taboos mean that people don't often talk about death. This lack of shared storytelling around potential afterlives can contribute to feelings of fear and anxiety. In this 90-minute workshop by Henrietta Lang, you'll be encouraged to imagine life after death in a creative way, connecting to a deeper sense of what this might mean to you. No one can 'know' what it's like, so why not imagine your own afterlife, as our ancestors did?

FRIDAY, OCTOBER 30TH • 09:30-11:00
SUNDAY, NOVEMBER 1ST • 13:30-15:00

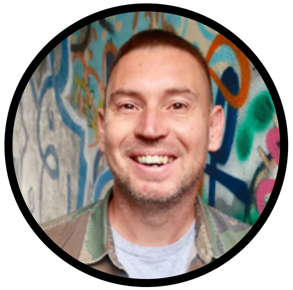

MARK LEMON PRESENTS...GRIEF CHAT

Our Grief Chats are intimate conversations between two guests who have both experienced the same type of grief or loss. They are hosted by writer and broadcaster Mark Lemon, who's behind the acclaimed podcast, Grief is my Superpower.

FRIDAY, OCTOBER 30TH • FROM 09:00

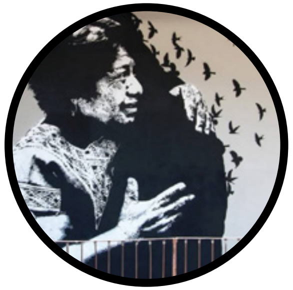

REFLECTIONS ON DEATH + DYING WITH RACHEL CLARKE, KATHRYN MANNIX + SAM GUGLANI

Why do we struggle to talk about death and how does that keep us from living? Does our discomfort around death impact on society and health? In this session we'll join a conversation with oncologist Sam Guglani and palliative care doctors Kathryn Mannix and Rachel Clarke as they consider death and dying.

SUNDAY, NOVEMBER 1ST • 17:00-18:00

HIGHLIGHTS

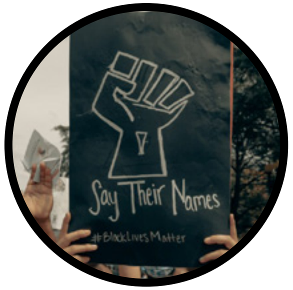

GRIEVING FOR JUSTICE

In a world rife with injustice, how can we make space for collective grief? Can grief be a force for positive change? How can we be more sensitive to the grief of those who have been silenced? We welcome Patrick Vernon, who kickstarted the campaign for an amnesty for the Windrush Generation, the Rt Hon. Stuart Lawrence, who runs the Stephen Lawrence Trust, and Kathryn de Prudhoe of the Covid-19 Bereaved Families for Justice group.

FRIDAY, OCTOBER 30TH • 12:30-13:30

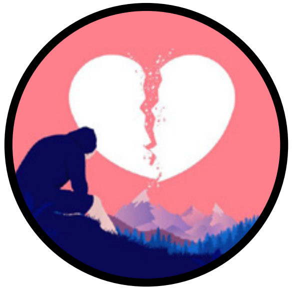

AM I GRIEVING RIGHT?

There is no one correct way to grieve, with as many ways to express grief as there are bereaved people. Join our panel of grief experts as they come together to bust some classic myths surrounding grief, seeking to enlighten us in the process.

FRIDAY, OCTOBER 30TH • 11:00-12:00

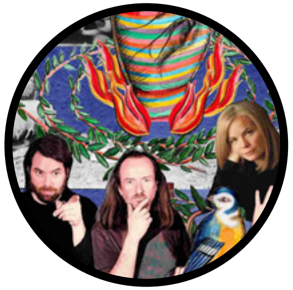

TUNNG PRESENTS... DEAD CLUB

Sam Genders and Becky Jacobs from the band Tunng will join Kaptin Barrett, Head of Music at Boomtown Festival, to discuss the band's extraordinary new work, Dead Club. In addition to their new album, Tunng's Dead Club project has created a podcast series speaking to those who work in the field of death including Derren Brown, Alain de Botton, Speech Debelle, Kathryn Mannix and AC Grayling.

SUNDAY, NOVEMBER 1ST • 18:00-19:00

THE LANGUAGE OF LOSS: POETRY, STORYTELLING + MUSIC

Join established and emerging poets and musicians including Brook Tate, Josie Alford, Vanessa Kisuule and Solomon O.B in this rare opportunity to both mourn and celebrate all that has been and all that is to arrive. The evening will be curated by poet, performer and writer, Rebecca Tantony.

FRIDAY, OCTOBER 30TH • 18:30-20:00

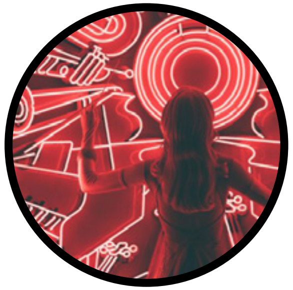

THE SOFA SINGERS

Join Good Grief's special edition of the Sofa Singers, adding your voice to hundreds of others as we sing Israel Kamakawiwo'ole's beautiful version of Over the Rainbow (find it here on YouTube). This will be an opportunity to pay tribute to those you've loved and lost and those who have died during the Covid-19 pandemic.

SATURDAY, OCTOBER 31ST • 11:15-12:15

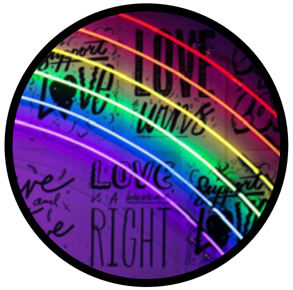

PANEL TALKS

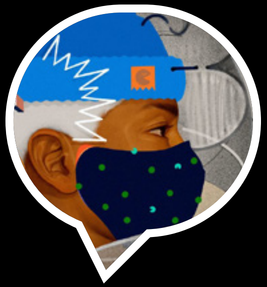

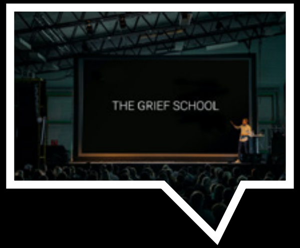

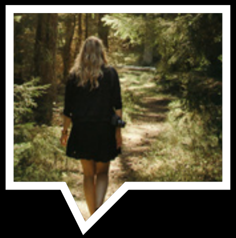

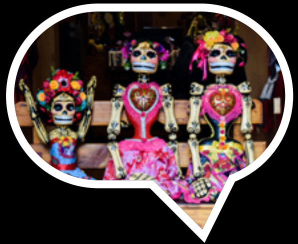

FRIDAY 30 OCTOBER

Is This The Age of Grief? **10:00-11:00**

People across the world appear to be surrounded by a pervasive sense of loss.
Are we entering the Age of Grief?

Am I Grieving Right? **11:00-12:00**

There is no single correct way to grieve, but as many ways to express grief as there are bereaved people. Join our panel of grief experts as they come together to bust some classic myths surrounding grief, seeking to enlighten us in the process.

Grieving for Justice **12:30-13:30**

Living in a world rife with injustice, how can we make space for collective grief? Can grief be a force for positive change? How can we be more sensitive to the grief of those who have been silenced?

The Covid Cataclysm - How do we Grieve for Normal? **14:30-15:30**

How do we grieve for the loss of normal life and all of our hopes, plans and expectations for the future? Join our experts as they discuss what many people are trying to get to grips with to during the pandemic: collective grief.

The Art of Pilgrimage - Going Back to Move Forward **15:30-16:30**

Revisiting the places where we feel connected to those we've lost can offer profound healing. Going back can be a powerful way of ultimately moving forwards, as this exploration of pilgrimage in relation to grief shows.

PANEL TALKS

SATURDAY 31 OCTOBER

Grieving for our Planet **10:00-11:00**

During this conversation about climate grief, Ed Gillespie, Anouchka Grose and Judith Anderson will discuss the rising tide of eco-anxiety and grief, as well as children's and adults' growing concerns about the climate crisis.

Empty Bed Blues - Losing a Life Partner **12:30-13:30**

This talk is essential viewing for anyone who has lost a partner, and for those supporting people who've lost partners.

Grief at the Kitchen Table **14:30-15:30**

Writers Nikesh Shukla, Valentine Warner and Olivia Potts consider how food can be a uniting factor in facing and moving through grief.

Over My Dead Body **15:30-16:30**

Many of us have a deeply uncomfortable relationship with dead bodies. Join Professor Alice Roberts and guests as they discuss the separation between the practical aspects of disposing of a dead body and the desire to grieve and memorialise a life.

The Dead Parent Club **17:00-18:00**

Cariad Lloyd and guests will delve deep into what it means to lose a parent, how early bereavement is processed at different stages of life and how it's possible to find meaning that drives you forward.

SUNDAY 1 NOVEMBER

The Grief Gift - Finding Meaning After Loss **12:00-13:00**

Grief can be a powerful catalyst for change. In this conversation, our panellists will discuss finding new purpose and meaning after losing someone dear to them.

The Healing Power of Nature in Grief **15:30-16:30**

Nature has an incredible ability to comfort and heal when we experience loss. It connects us with the cycles of the seasons and allows us to see the world, our lives, and ourselves in new ways.

Reflections on Death + Dying with Rachel Clarke, Kathryn Mannix and Samir Guglani **17:00-18:00**

Why do we struggle to talk about death and how does that keep us from living? How does our discomfort with death and dying impact on society and our health?

WORKSHOPS + WEBINARS

FRIDAY 30 OCTOBER

Making Friends with your Afterlife
09:30-11:00 — Webinar

Grief in Shakespeare
11:15-12:15 — Webinar

Writing Poetry for Lost Loved Ones
12:45-14:15 — Webinar

How to Support Someone Who's Bereaved
14:45-15:45 — Webinar

Creating Meaningful Funerals + Memorial Ceremonies
16:45-17:15 — Webinar

Good Grief Trust - Grief Cafe
17:30-18:30 — Workshop

SATURDAY 31 OCTOBER

Feel Into It - Yoga to Explore Feelings
09:00-10:00 — Workshop

Self Counselling Through Art with Gary Andrews
10:15-11:15 — Webinar

Writing Memoir to Heal with Nikesh Shukla
11:30-12:30 — Webinar

Death Cafe
12:45-13:45 — Workshop

Life's Questions - Talking to Young People about Grief
15:30-16:30 — Webinar

Virginia Woolf and the Literature of Loss
16:45-17:45 — Webinar

Grief + Philosophy
18:00-18:45 — Webinar

SUNDAY 1 NOVEMBER

Feel Into It - Yoga to Explore Feelings
09:30-10:30 — Workshop

Grief in Literature
11:00-12:00 — Webinar

Making Friends with Your Afterlife
13:30-15:00 — Webinar

Grief in Film
15:15-16:15 — Webinar

The Human Memorial
17:00-18:00 — Webinar

INTERVIEWS

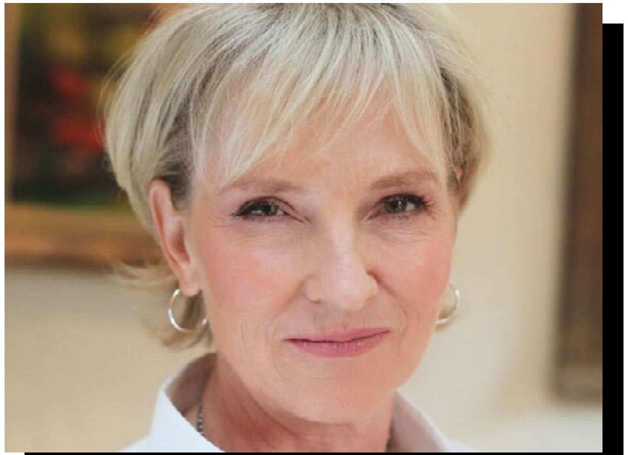

JULIA SAMUEL: WHEN THE WORST HAPPENS - COPING WITH GRIEF + CRISIS

FRI, OCT 30TH • 17:00-17:45

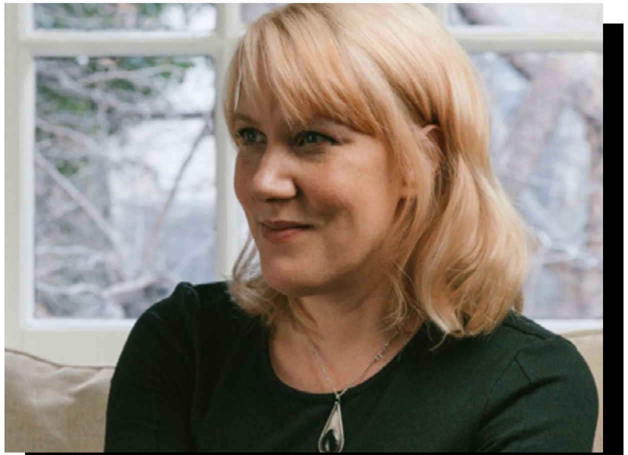

ELAINE KASKET: DIGITAL DEATH, ONLINE AFTERLIFE

SAT, OCT 31ST • 13:30-14:30

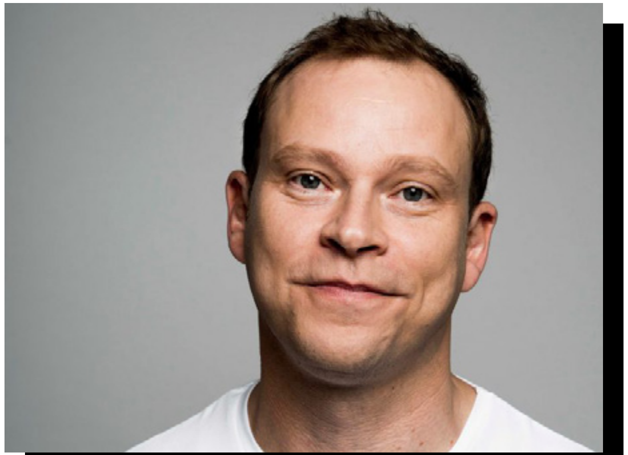

ROBERT WEBB INTERVIEW

SAT, OCT 31ST • 18:00-18:45

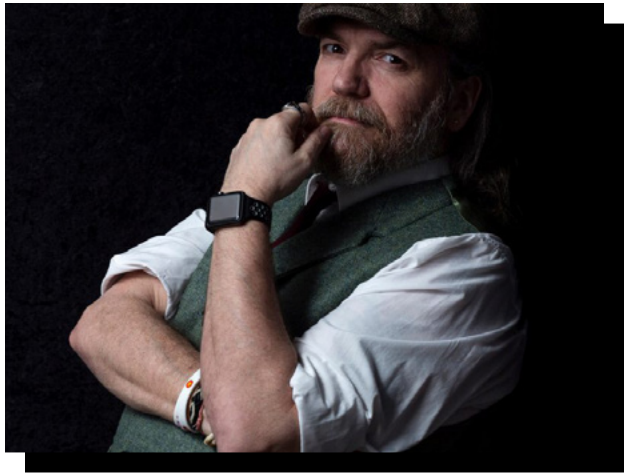

GARY ANDREWS ON FINDING JOY

SUN, NOV 1ST • 10:00-10:45

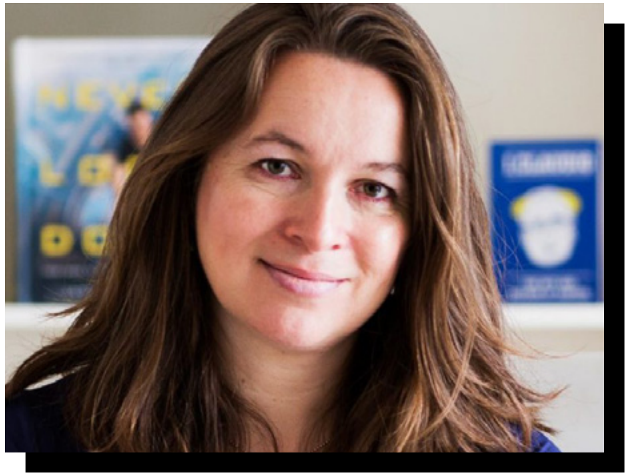

RACHEL CLARKE: A DOCTOR'S STORY OF LOVE + LOSS

SUN, NOV 1ST • 14:30-15:15

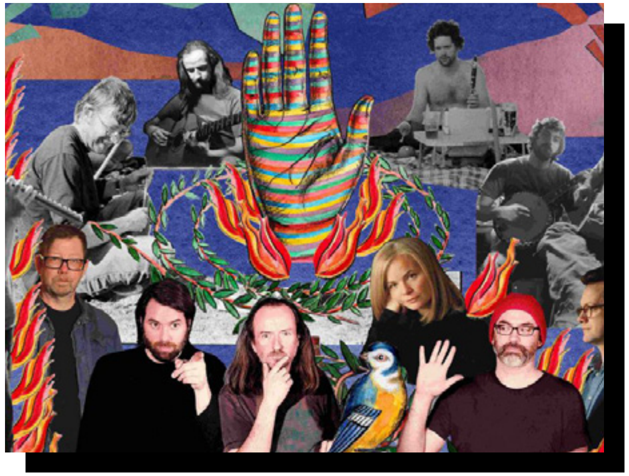

TUNNG PRESENTS... DEAD CLUB

SUN, NOV 1ST • 18:00-19:00

GRIEF CHATS

Each Grief Chat will repeat on a loop from 09:00 on Oct 30th until 19:00 on Nov 1st on the Grief Chat pages of our website. After this they'll be part of our on-demand subscription.

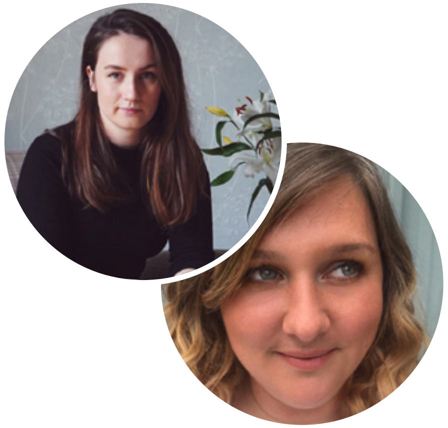

ALEXA + JEN
LAUGHING THROUGH GRIEF
Comedians Alexa and Jen use humour to confront their grief.

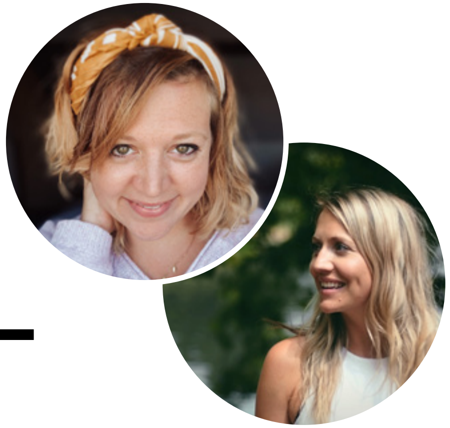

KAREN + KIM
DEATH BY SUICIDE
Karen and Kim reflect on losing a loved one to suicide and turning their grief outwards to help others.

GARY + DAVID
SEPSIS
Gary and David talk about losing a loved one suddenly to sepsis.

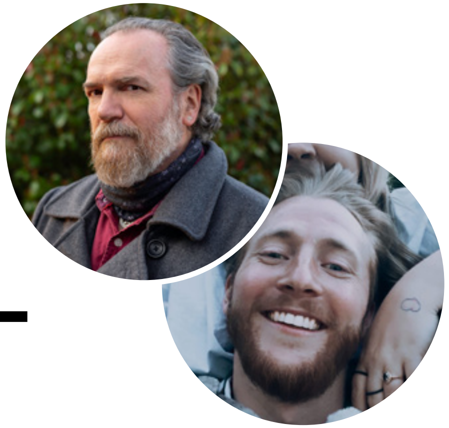

JANE + GILL
THE DEATH OF A CHILD
Jane and Gill's sons both died suddenly, aged 22, when travelling abroad.

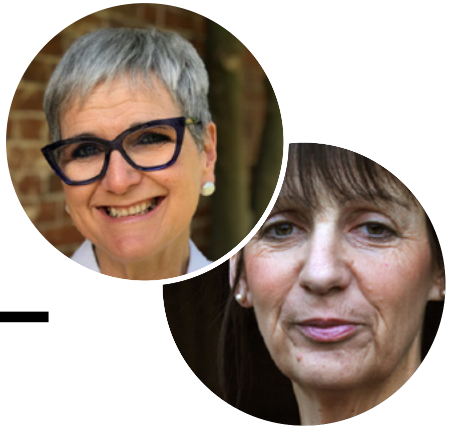

GRIEF CHATS

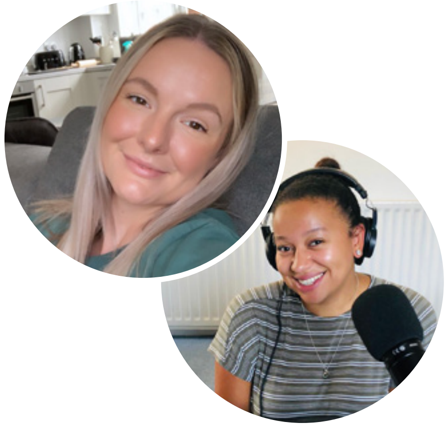

AMBER + KATHRYN
GRIEF PODCASTERS
Amber and Kathryn both lost their mums when they were teenagers and now create grief podcasts to support young people.

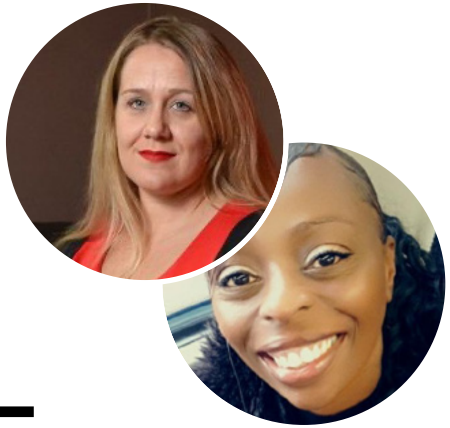

ALISON + RACHEL
KNIFE CRIME
Alison and Rachel's sons were both victims of knife crime.

ANN + OLIVIA
CANCER
Both Ann and Olivia's families have been torn apart by cancer. Hear about the support they offer to other bereaved young people on Instagram.

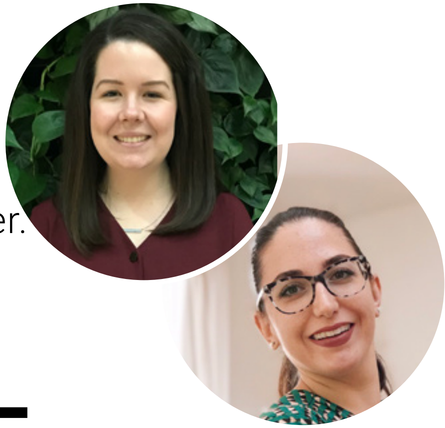

LIAM + CALLUM
MALE GRIEF
Callum and Liam from Let's Talk About Loss discuss the challenges faced by men who need to grieve.

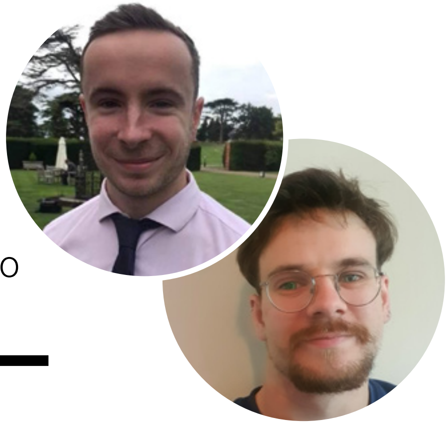

GRIEF SCHOOL

Some people believe there are as many as 44 different types of grief. At our Grief School, we'll be delving into some of the ways we experience loss, so that we can help people to better understand the nature and uniqueness of their grief. Each session will be facilitated by a bereavement organisation and will feature academic researchers or authors, as well as 'experts through experience' - people who have been personally affected by each type of bereavement or loss.

Each Grief School event will be repeated on a loop will be available on a loop from 09:00 on Oct 30th until 19:00 on Nov 1st on the Grief School pages of our website. After this they'll be part of our £20 on-demand subscription package.

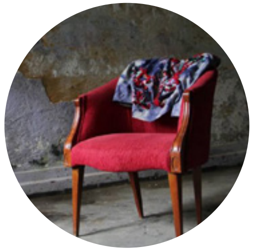

SUICIDE

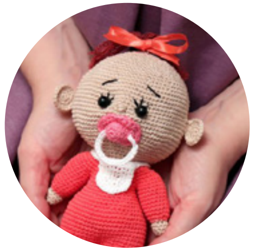

INFERTILITY + BABY LOSS

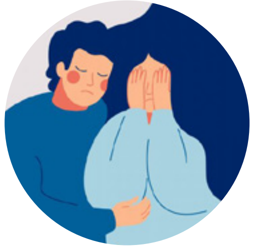

STILLBIRTH

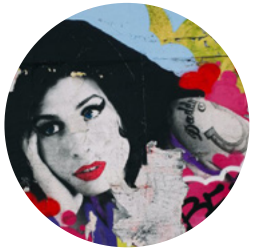

SUBSTANCE MISUSE

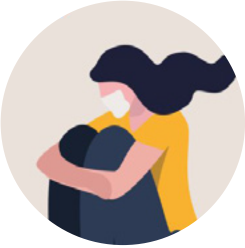

LIFE-THREATENING ILLNESS

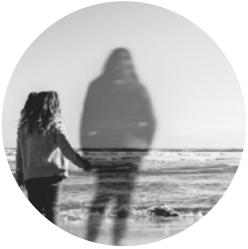

CHILDHOOD BEREAVEMENT

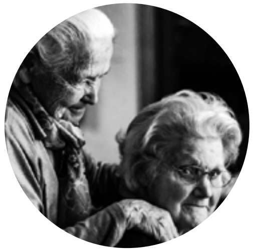

DEMENTIA

GRIEF SCHOOL

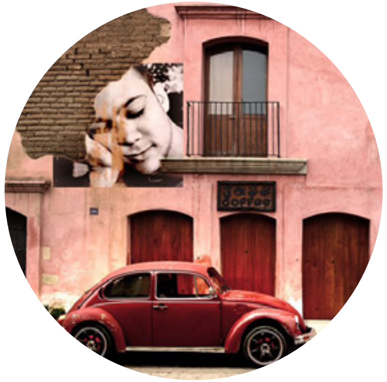

THE DEATH
OF A CHILD

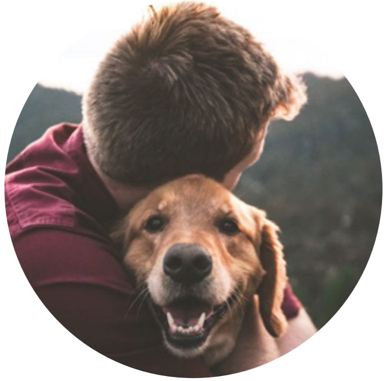

PET LOSS

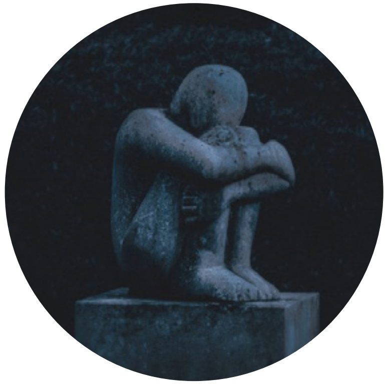

DELAYED
GRIEF

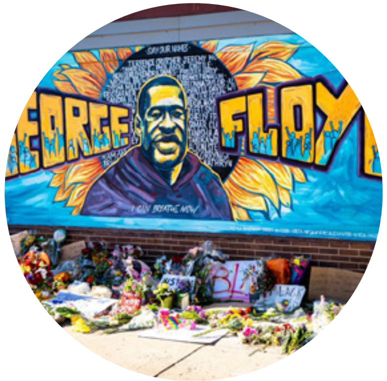

**TRAUMATIC
LOSS**

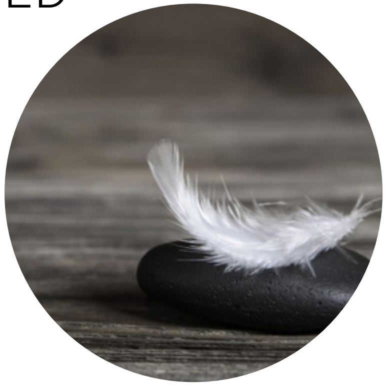

DUAL PROCESS
MODEL OF
GRIEF

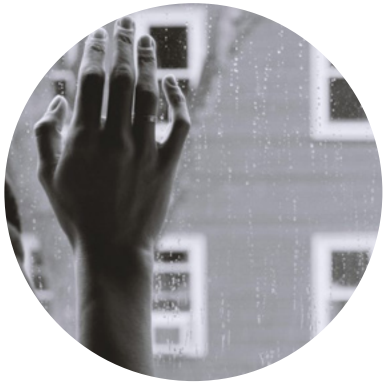

**GRIEVING DURING
COVID-19**

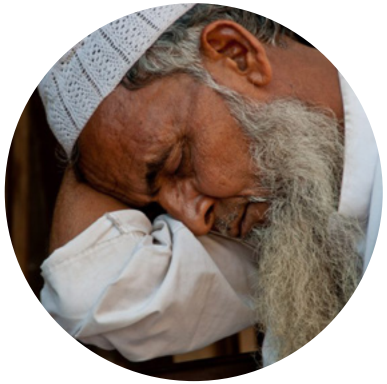

MUSLIM GRIEF
AND BEREAVEMENT

GOOD GRIEF
A FESTIVAL
OF LOVE & LOSS

WWW.GOODGRIEFFEST.COM

@GoodGriefFest @goodgrieffestival @GoodGriefFest

wellcome

University of BRISTOL

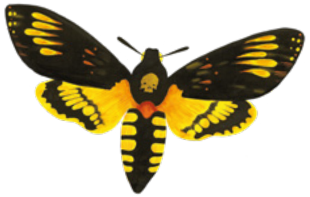

Supplement: sj-pdf-3-pcr-10.1177_26323524231189523 – Supplemental material for Engaging and supporting the public on the topic of grief and bereavement: an evaluation of Good Grief Festival [file sj-pdf-3-pcr-10.1177_26323524231189523.pdf]
