# Supplementary material for: Engaging and supporting the public on the topic of grief and bereavement: an evaluation of Good Grief Festival
Source: Palliat Care Soc Pract. 2023 Jul 30;17:26323524231189523. doi: 10.1177/26323524231189523 (PMC10392217; doi:10.1177/26323524231189523)
Supplement: sj-pdf-4-pcr-10.1177_26323524231189523 – Supplemental material for Engaging and supporting the public on the topic of grief and bereavement: an evaluation of Good Grief Festival [file sj-pdf-4-pcr-10.1177_26323524231189523.pdf]

GOOD GRIEF

A VIRTUAL FESTIVAL OF LOVE & LOSS

30 OCTOBER - 1 NOVEMBER 2020

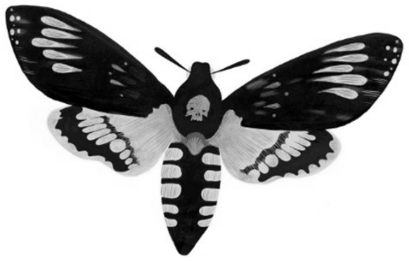

Thank you for completing this 2-minute survey

The information you provide will help us understand the reach and impact of the festival. Responses are anonymous. Please complete the survey only once, even if you are attending multiple events.

By participating in this survey you agree that your responses may be used in future reports about the festival. All data collected in this survey will be held securely at the University of Bristol in accordance with the General Data Protection Regulations (GDPR; EU 2016/679). For more information, please contact lucy.selman@bristol.ac.uk.

* 1. I am attending the festival to (tick all that apply):

- [ ] Be inspired
- [ ] Feel part of a like-minded community
- [ ] Share or express experiences
- [ ] Learn about grief and bereavement
- [ ] Find out about local bereavement support
- [ ] Other (please specify)

2. Have you experienced the death of a relative, partner or close friend (otherwise known as bereavement)?

- ○ Yes, within the last year
- ○ Yes, within the last 5 years
- ○ Yes, more than 5 years ago
- ○ No

3. To what extent do you agree or disagree, if at all, with the following statements about talking to someone who has recently experienced the death of a relative, partner or close friend.

	Strongly agree	Tend to agree	Tend to disagree	Strongly disagree	Don't know
I would be scared of ‘saying the wrong thing’ to someone who was recently bereaved	◯	◯	◯	◯	◯
I would avoid talking to someone who was recently bereaved about their bereavement because I wouldn’t know how to help	◯	◯	◯	◯	◯
I would know what to do if someone who was recently bereaved told me they were having trouble	◯	◯	◯	◯	◯
I would know what kind of help or support to offer someone who was bereaved	◯	◯	◯	◯	◯

4. Which of the following best describes you?

- ◯ Member of the public
- ◯ Bereavement counsellor
- ◯ Academic interested in grief
- ◯ Clinician
- ◯ Teacher
- ◯ Student
- ◯ Other (please specify)

[ ]

* 5. How old are you?

- ◯ Under 18
- ◯ 18-24
- ◯ 25-34
- ◯ 35-44
- ◯ 45-54
- ◯ 55-64
- ◯ 65-74
- ◯ 75 or over

6. Which of the following describes how you think of yourself? Please select one option.

- ◯ Male
- ◯ Female
- ◯ In another way:

7. What is your ethnic group?

- ◯ White
- ◯ Mixed/Multiple ethnic groups
- ◯ Asian/Asian British
- ◯ Black/African/Caribbean/Black British
- ◯ Other ethnic group:

8. In what country do you currently reside?

- ◯ United Kingdom
- ◯ Other (please specify)

* 9. What is your postcode? (This is to understand which areas attendees come from)
