# Supplementary material for: Engaging and supporting the public on the topic of grief and bereavement: an evaluation of Good Grief Festival
Source: Palliat Care Soc Pract. 2023 Jul 30;17:26323524231189523. doi: 10.1177/26323524231189523 (PMC10392217; doi:10.1177/26323524231189523)
Supplement: sj-pdf-5-pcr-10.1177_26323524231189523 – Supplemental material for Engaging and supporting the public on the topic of grief and bereavement: an evaluation of Good Grief Festival [file sj-pdf-5-pcr-10.1177_26323524231189523.pdf]

GOOD GRIEF

A VIRTUAL FESTIVAL OF LOVE & LOSS

30 OCTOBER - 1 NOVEMBER 2020

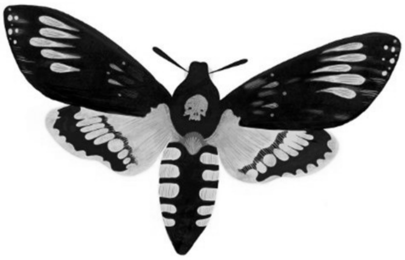

Thank you for completing this short feedback survey

The information you provide will help us understand the reach and impact of the festival. Responses are anonymous. Please complete the survey only once, even if you attended multiple events.

By participating in this survey you agree that your responses may be used in future reports about the festival. All data collected in this survey will be held securely at the University of Bristol in accordance with the General Data Protection Regulations (GDPR; EU 2016/679). For more information, please contact lucy.selman@bristol.ac.uk.

* 1. How many festival events did you attend?

- [ ] 1 or 2
- [ ] 3-5
- [ ] A whole day
- [ ] Several days
- [ ] I did not attend in the end - please tell us why (and then skip to question 7)

2. At the event(s) I attended I (tick all that apply):

- [ ] Was inspired
- [ ] Felt part of a like-minded community
- [ ] Shared or expressed my experiences
- [ ] Learnt about grief and bereavement
- [ ] Found out about local bereavement support
- [ ] Other (please specify)

3. To what extent do you agree with the following statement: Through attending the festival I feel more confident talking about grief.

- [ ] Strongly agree
- [ ] Tend to agree
- [ ] Neither agree nor disagree
- [ ] Tend to disagree
- [ ] Strongly disagree
- [ ] Don't know
- [ ] Not applicable - I already felt as confident as I could.

4. How would you rate your experience of the festival overall?

- [ ] 0 = poor
- [ ] 1
- [ ] 2
- [ ] 3
- [ ] 4
- [ ] 5 = excellent

5. Please tell us your suggestions for how the festival could have been improved.

6. Is there anything else you would like to say about the festival?

7. To what extent do you agree or disagree, if at all, with the following statements about talking to someone who has recently experienced the death of a relative, partner or close friend.

	Strongly agree	Tend to agree	Tend to disagree	Strongly disagree	Don't know
I would be scared of ‘saying the wrong thing’ to someone who was recently bereaved	◯	◯	◯	◯	◯
I would avoid talking to someone who was recently bereaved about their bereavement because I wouldn’t know how to help	◯	◯	◯	◯	◯
I would know what to do if someone who was recently bereaved told me they were having trouble	◯	◯	◯	◯	◯
I would know what kind of help or support to offer someone who was bereaved	◯	◯	◯	◯	◯

8. Which of the following best describes you?

- ◯ Member of the public
- ◯ Bereavement counsellor
- ◯ Academic interested in grief
- ◯ Clinician
- ◯ Teacher
- ◯ Student
- ◯ Other (please specify)

* 9. How old are you?

- ◯ Under 18
- ◯ 18-24
- ◯ 25-34
- ◯ 35-44
- ◯ 45-54
- ◯ 55-64
- ◯ 65-74
- ◯ 75 or over

10. Which of the following describes how you think of yourself? Please select one option.

- ◯ Male
- ◯ Female
- ◯ In another way:

11. What is your ethnic group?

- ◯ White
- ◯ Mixed/Multiple ethnic groups
- ◯ Asian/Asian British
- ◯ Black/African/Caribbean/Black British
- ◯ Other ethnic group:

12. In what country do you currently reside?

- ◯ United Kingdom
- ◯ Other (please specify)

* 13. What is your postcode? (This is to understand which areas attendees come from)

14. We will be conducting a small number of focus groups about the festival. If you might be willing to take part, please enter your email address below. Your email address will not be shared or used for any other purpose.
